# Lipopolysaccharide O structure of adherent and invasive *Escherichia coli* regulates intestinal inflammation via complement C3

**DOI:** 10.1371/journal.ppat.1008928

**Published:** 2020-10-07

**Authors:** Masashi Ohno, Mizuho Hasegawa, Atsushi Hayashi, Gustavo Caballero-Flores, Christopher J. Alteri, Trevor D. Lawley, Nobuhiko Kamada, Gabriel Núñez, Naohiro Inohara

**Affiliations:** 1 Department of Pathology, University of Michigan Medical School, Ann Arbor, Michigan, United States of America; 2 Department of Medicine, Shiga University of Medical Science, Otsu, Japan; 3 Internal Medicine, University of Michigan Medical School, Ann Arbor, Michigan, United States of America; 4 Miyarisan Pharmaceutical Co., Ltd., Central Research Institute, Saitama, Japan; 5 Rogel Cancer Center, University of Michigan Medical School, Ann Arbor, Michigan, United States of America; 6 Department of Natural Sciences, University of Michigan-Dearborn, Dearborn, Michigan, United States of America; 7 Wellcome Sanger Institute, Wellcome Genome Campus, Hinxton, United Kingdom; University of California Davis School of Medicine, UNITED STATES

## Abstract

Gut dysbiosis associated with intestinal inflammation is characterized by the blooming of particular bacteria such as adherent-invasive *E*. *coli* (AIEC). However, the precise mechanisms by which AIEC impact on colitis remain largely unknown. Here we show that antibiotic-induced dysbiosis worsened chemically-induced colitis in IL-22-deficient mice, but not in wild-type mice. The increase in intestinal inflammation was associated with the expansion of *E*. *coli* strains with genetic and functional features of AIEC. These *E*. *coli* isolates exhibited high ability to out compete related bacteria via colicins and resistance to the host complement system *in vitro*. Mutation of *wzy*, the lipopolysaccharide O polymerase gene, rendered AIEC more sensitive to the complement system and more susceptible to engulfment and killing by phagocytes while retaining its ability to outcompete related bacteria in vitro. The wzy AIEC mutant showed impaired fitness to colonize the intestine under colitic conditions, but protected mice from chemically-induced colitis. Importantly, the ability of the *wzy* mutant to protect from colitis was blocked by depletion of complement C3 which was associated with impaired intestinal eradication of AIEC in colitic mice. These studies link surface lipopolysaccharide O-antigen structure to the regulation of colitic activity in commensal AIEC via interactions with the complement system.

## Author summary

Susceptibility to colitis is associated with abnormal changes in microbial populations of the gut in individuals with impaired immune systems. A common change is the increased abundance of adherent-invasive *Escherichia coli* (AIEC), a bacterial population that is associated with the development of inflammatory bowel disease. However, the precise mechanisms by which AIEC influences colitis remain poorly understood. Here we show that changes in the microbial population induced by administration of antibiotics include a bloom of *Escherichia coli*, which was associated with worsening chemically-induced colitis in IL-22-deficient mice. *E*. *coli* strains isolated from the gut of such mice exhibited AIEC features, high ability to outcompete related bacteria and resistance to the host complement system *in vitro*. Mutation of the gene required for the biosynthesis of lipopolysaccharide O side chains rendered AIEC sensitive to complement-mediated elimination in colitic mice. Upon oral inoculation, the mutant AIEC protected the mice from colitis. Our study provides new insights into the mechanisms by which gut microbes control colitis via the complement system and a possible strategy for the generation of probiotic strains resistant to host elimination.

## Introduction

The surface of bacterial symbionts and pathogens is important for the interaction of these microorganisms with their hosts. On the cell surface, microbes express various conserved molecules, structures and antigens that can be recognized by the host immune system for microbe killing and eradication [1]. Conversely, many microbes can evade host recognition through the production of cell surface proteins and polysaccharides including the capsule (K-antigen) and lipopolysaccharide side chains (O-antigen) structures in Enterobacteriaceae [2, 3]. These bacterial polysaccharides confer resistance to the host immune system by inhibiting the binding of antibodies to microbial surface antigens and impairing complement-mediated phagocytosis [4, 5]. Thus, structural variation in surface polysaccharides produced by bacteria can affect the survival of microorganisms that invade host tissues as well as the susceptibility of the host to bacteremia and sepsis [6]. Extraintestinal pathogenic Escherichia coli (ExPEC) strains produce specific K or O serotypes and are often resistant to host systemic immunity [7, 8], while intestinal pathogenic *E*. *coli* (InPEC) strains can cause or promote colitis, diarrhea and inflammatory bowel disease (IBD) through their ability to interact with intestinal cells [9]. These host/bacterial interactions are primarily mediated by bacterial adhesive molecules and toxins, but the role of surface polysaccharides in the regulation of intestinal inflammation remains largely unknown.

Adherent-invasive *E*. *coli* (AIEC) is a group of InPEC that is associated with IBD including Crohn’s disease (CD) and ulcerative colitis (UC) [10, 11]. AIEC expresses surface adhesins including type I fimbriae that are important for its ability to interact with host cells and for biofilm formation [12]. Structural variation of FimH in type I fimbria is associated with higher ability of AIEC to adhere to and invade host cells [13]. Furthermore, the invasiveness properties of AIEC have been suggested to be important for evading recognition and elimination by the host immune system [14, 15]. Collectively, these observations suggest that the resistance of AIEC to host recognition and elimination impacts on bacterial survival and the ability of AIEC to promote intestinal inflammation. However, the mechanisms by which AIEC evades the host immune system to promote bacterial survival and AIEC-associated colitis remain unknown.

Multiple lines of evidence suggest that IBD is caused by genetic and environmental factors that alter intestinal homeostasis in genetically susceptible individuals [16]. Many IBD-associated loci are known to regulate the recognition and killing of bacteria as well as the function of immune cells [17]. For example, *IL23R* and *TNFSF15* that are critical for the activation of innate lymphoid cells 3 (ILC3) and Th17 cells, cell populations that are important for the maintenance of the intestinal barrier and tissue repair, are associated with IBD susceptibility [18, 19]. ILC3 and Th17 cells produce IL-22, a cytokine that mediates protective immune responses in infectious and chemically-induced colitis[20]. Upon enteric bacterial infection, IL-22 is produced locally in the intestine, but can also reach the liver where it acts on hepatocytes to induce the secretion of acute phase proteins including complement protein C3 [6, 21]. In patients with IBD and animal models of colitis, there is an alteration in the composition and structure of the gut microbiota which includes a bloom of AIEC [22, 23]. Furthermore, colonization by certain AIEC strains can promote the induction of colitis in IL10-deficient mice [24]. In the current studies, we used *Il22*^-/-^ mice to study the role of surface lipopolysaccharide in the regulation of AIEC colonization and AIEC-associated colitis.

## Results

### Bacterial dysbiosis induced by antibiotic treatment enhances DSS-induced colitis in the absence of IL-22

**Fig 1:**
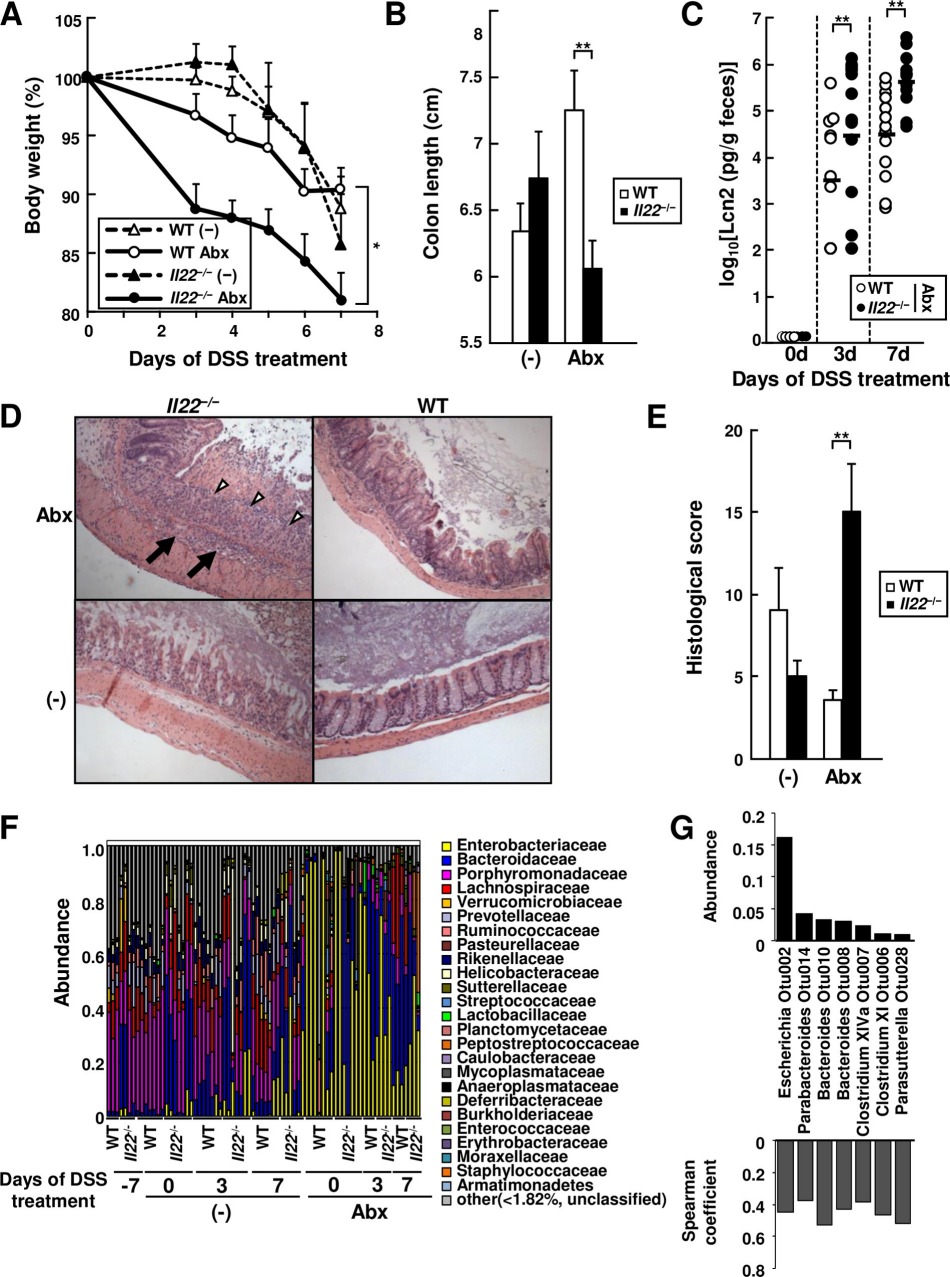
Bacterial dysbiosis induced by antibiotic treatment regulates DSS-induced colitis in IL-22 deficient mice. WT and *Il22*^*−/−*^ mice were pretreated with the antibiotic cocktail (Abx) or mock (-) for six days after co-housing. Mice were given 2.5% DSS in the drinking water. Mice were monitored for body weight (A, n = 9 to 22 per group), colon length after 7 days of DSS treatment and (B) fecal lipocalin-2 (Lcn2) levels (C) on the indicated days of DSS treatment. (D and E) representative histology of hematoxylin and eosin (HE)–stained colonic sections (D) and histological scores (E) of colons from indicated mice 7 days after DSS treatment (n = 4–7 per group). Open arrowheads and solid arrows indicate mucosal ulcer with total loss of epithelium, and edema with submucosal inflammation, respectively. (F) Taxonomic microbiota composition at the family level in mice before antibiotic treatment (day -7), on day 6 after antibiotic treatment (day 0 of DSS treatment), and on day 3 and 7 after DSS treatment (n = 3 to 7). (G) *Escherichia* as the most abundant bacteria that are associated with Lcn2 levels during DSS treatment. The bacteria that showed significant association (p< 0.05) by Spearman's rank correlation analysis with OTU abundance and fecal lipocalin 2 levels in colitic intestine of DSS-treated mice. Error bars represent SEM. *p < .05, **p < .01.

We used a chemically-induced colitis model in which oral administration of dextran sulfate sodium (DSS) triggers epithelial damage and inflammation to investigate the role of the microbiota in colitis. To address the role of dysbiosis-associated colitis in the immunocompromised host, we used mice lacking IL-22, a cytokine that is important for immune protection at the intestinal barrier [25]. WT and *Il22*^*−/−*^ mice were pretreated with a cocktail of antibiotics for 6 days to induce dysbiosis prior to the administration of DSS. We assessed colitis by measuring body weight, colon length, histopathology and fecal lipocalin-2 (Lcn2), a marker of intestinal inflammation [26]. We found that *Il22*^*−/−*^ mice pretreated with antibiotics showed more weight loss, more shortening of colon length, and greater fecal lipocalin-2 levels after DSS treatment than WT mice pretreated with antibiotics, and untreated WT or *Il22*^*−/−*^ mice (**Fig 1A to 1C**). Consistent with these findings, higher pathology scores were found in the intestine of antibiotic-pretreated *Il22*^*−/−*^ mice than in antibiotic-treated WT mice and untreated mice (**Figs 1D, 1E and S1A**). These results suggest that antibiotic-induced dysbiosis exacerbates DSS-induced colitis in the absence of IL-22.

To determine how antibiotic treatment affects the microbiota during DSS-induced colitis, we analyzed the composition of the microbiota using Illumina MiSeq 16S rRNA gene sequencing. Consistent with previous studies [6, 27], treatment of *Il22*^*−/−*^ and WT mice with a cocktail of 7 antibiotics resulted in an increased abundance of Enterobacteriaceae before and after treatment with DSS (**Fig 1F**). The dominance of Enterobacteriaceae in antibiotic-treated *Il22*^*−/−*^ and WT mice was accompanied by an increase in Bacteroidaceae after DSS treatment (**Fig 1F**). We confirmed that antibiotic treatment increases the abundance of Enterobacteriaceae in both *Il22*^*−/−*^ and WT mice by quantitative PCR using specific primers (**S1B Fig**). To identify bacteria associated with colitis, we determined the most dominant bacteria that are associated with increased fecal Lcn2 levels by Spearman ranking correlation analysis. We found that *Escherichia* was the most abundant genera associated with increased Lcn2 levels in the intestine (Spearman's ranking correlation coefficient = 0.449, p<0.0014, average abundance = 16.2%) (**Fig 1G**). These results suggest that the blooming of certain Enterobacteriaceae species including *E*. *coli* is associated with antibiotic-induced dysbiosis in WT and IL-22-deficient mice. Furthermore, IL-22 protects the host from dysbiosis-associated colitis in DSS-treated animals.

### Colitis-associated *E*. *coli* isolates belong to AIEC

To determine the role of Enterobacteriaceae that accumulate during antibiotic-induced dysbiosis in colitis, we isolated Enterobacteriaceae species from the intestine of antibiotic-treated *Il22*^*−/−*^ mice. Consistent with previous studies [27], bacteria belonging to *Enterobacter* and *Escherichia* were identified as dominant Enterobacteriaceae genera that bloom after treatment with antibiotics (**S1 Table**). To determine the role of these bacterial species in colitis, we isolated *E*. *coli* as the most abundant bacteria associated with increased Lcn2 levels in the intestine (**Fig 1G**), and determined full genomic sequences of four independent *E*. *coli* isolates, NI1429, NI1413, NI1423 and NI1522. Analysis of their genomes showed that all of four strains belong to the B2-phylogroup (ChuA^+^YjaA^+^TSPE4.C2^-^) which is common in human AIEC strains [13]. Furthermore, the four *E*. *coli* strains were genetically similar and showed high similarity to human AIEC strains when compared to ≈ 600 reference *E*. *coli* strains by Roary (**S2 Table, S2A Fig**). The four mouse *E*. *coli* isolates and human AIEC reference strains showed high homology in protein sequences (>91%) and their genomes contained a unique set of ≈ 340 genes not found in the reference *E*. *coli* laboratory strain K-12 MG1655 (**S2 Table**). These genes conserved in mouse and human AIEC strains include many putative virulence genes (**S2 Fig Panel B**) In addition, the genomes of mouse AIEC isolates harbor several genes encoding type I fimbrial proteins, invasin-like adhesins and strain-specific flagella (**S2 Table**). The polymorphisms of type I fimbrial protein FimH in the mouse isolates were identical to those reported to be important for adhesiveness and invasiveness in human AIEC [13]. These results suggest that *E*. *coli* isolated from colitic *Il22*^*−/−*^ mice possess genetic features that are characteristic of human AIEC.

**Fig 2:**
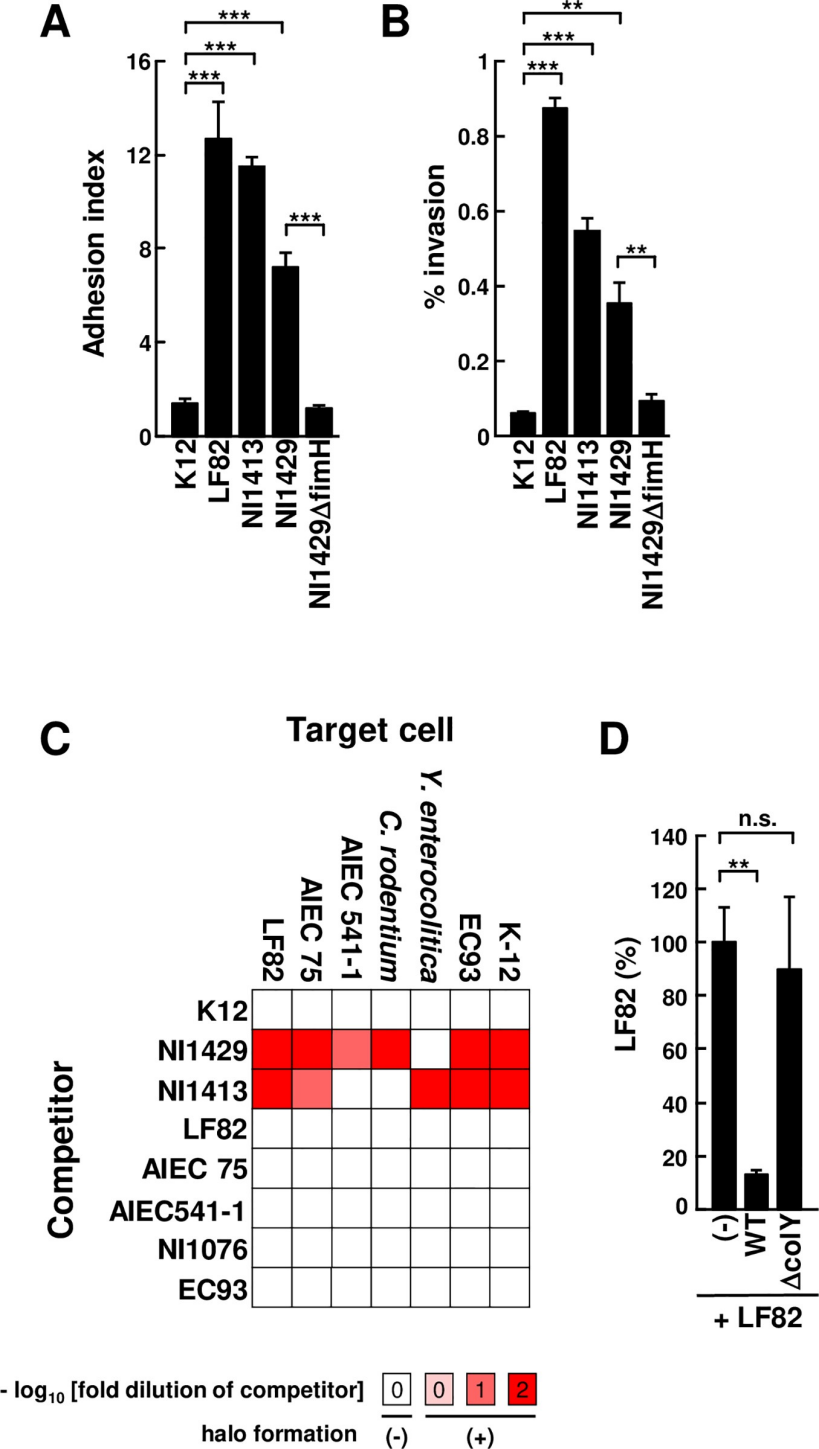
Colitis-associated *E*. *coli* isolates have AIEC features and possess high ability to outcompete related bacteria. T84 intestinal epithelial cells were infected with indicated bacteria at a MOI of 10. The number of adhered bacteria per cell (A) and the percentage of internalized bacteria compared with the number of initial bacteria (B). (C) Inhibitory effect of competitor strains was determined by inhibition halo assay on lawns of target strains. Red-filled boxes indicate halo formation while white-filled boxes indicate no halo formation in the presence of 0 to 2 log fold dilution of competitor strain. (D) LF82 was cultured alone or co-cultured with indicated bacteria at 1:1 for 3 hr, then the percentage of co-cultured LF82 compared with culture of LF82 alone was calculated. WT; NI1429Str, Δ*wzy*; NI1429StrΔ*wzy*, Δ*colY*; NI1429StrΔ*colY*. Error bars represent SEM. *p < .05, **p < .01, ***p < .001.

The genetic evidence that colitis-associated *E*. *coli* mouse isolates exhibit multiple features of AIEC prompted studies to assess whether these bacterial strains exhibit phenotypes characteristic of AIEC. To determine if the mouse *E*. *coli* isolates function as AIECs, we performed adhesion and invasion assays using T84 intestinal epithelial cells as described [28]. The colitis-associated *E*. *coli* isolates, NI1413 and NI1429 showed the ability to adhere to and invade into T84 intestinal epithelial cells (**Fig 2A and 2B**). To verify the role of Fim type I fimbrial adherence in the AIEC phenotype, we deleted *fimH* in one of the mouse isolates by homologous recombination. Consistent with the important role of the type I fimbriae in the AIEC phenotype of the human LF82 strain [13], the adhesiveness and invasiveness of NI1429 was lost after deletion of *fimH* (**Fig 2A and 2B**). Collectively, these results indicate that the murine isolates are AIECs.

### Colitis-associated AIEC possess high ability to outcompete symbiotic bacteria

Further evaluation of bacterial genomes of colitis-associated *E*. *coli* isolates revealed that they contain putative genes involved in inter-species competition. These include genes for the production of colicins on plasmids, type VI secretion system (T6SS) effectors and factors for contact-dependent growth inhibition (S2 **Fig** **Panel B**, **S2 Table**). These findings suggest that colitis-associated *E*. *coli* isolates have the potential to outcompete other bacteria and acquire dominance under dysbiotic conditions. Because all four colitis-associated *E*. *coli* isolates contained plasmid-encoded colicins that act as antimicrobial peptides against competitive bacteria [29], we performed halo assays to determine the ability of the isolates to outcompete other enterobacteria by overlaying the competitors on target bacteria [29]. We found that two colitis-associated *E*. *coli* strains, NI1413 and NI1429, exhibited potent ability to outcompete other *E*. *coli* strains and related enterobacteria, including human AIECs and the mouse pathogen *C*. *rodentium* (**Fig 2C**). To further characterize the role of colicins in bacterial competition, we deleted *colY*, a colicin gene in NI1429, a representative mouse AIEC isolate. In these experiments, we co-cultured ampicillin-resistant strain LF82, a reference human AIEC strain with streptomycin-resistant NI1429 derivatives (NI1429Str) including WT or an isogenic Δ*colY* mutant. Consistent with the results of the halo assay, WT NI1429Str exhibited robust ability to inhibit the growth of LF82 and the inhibitory activity was dependent on the presence of colicin Y (**Fig 2D**). Collectively, these results indicate colitis-associated *E*. *coli* are AIEC with high competitive ability against related enterobacteria.

### Intestinal AIEC colonization is regulated by the surface polysaccharide layer and IL-22 under colitic conditions

**Fig 3:**
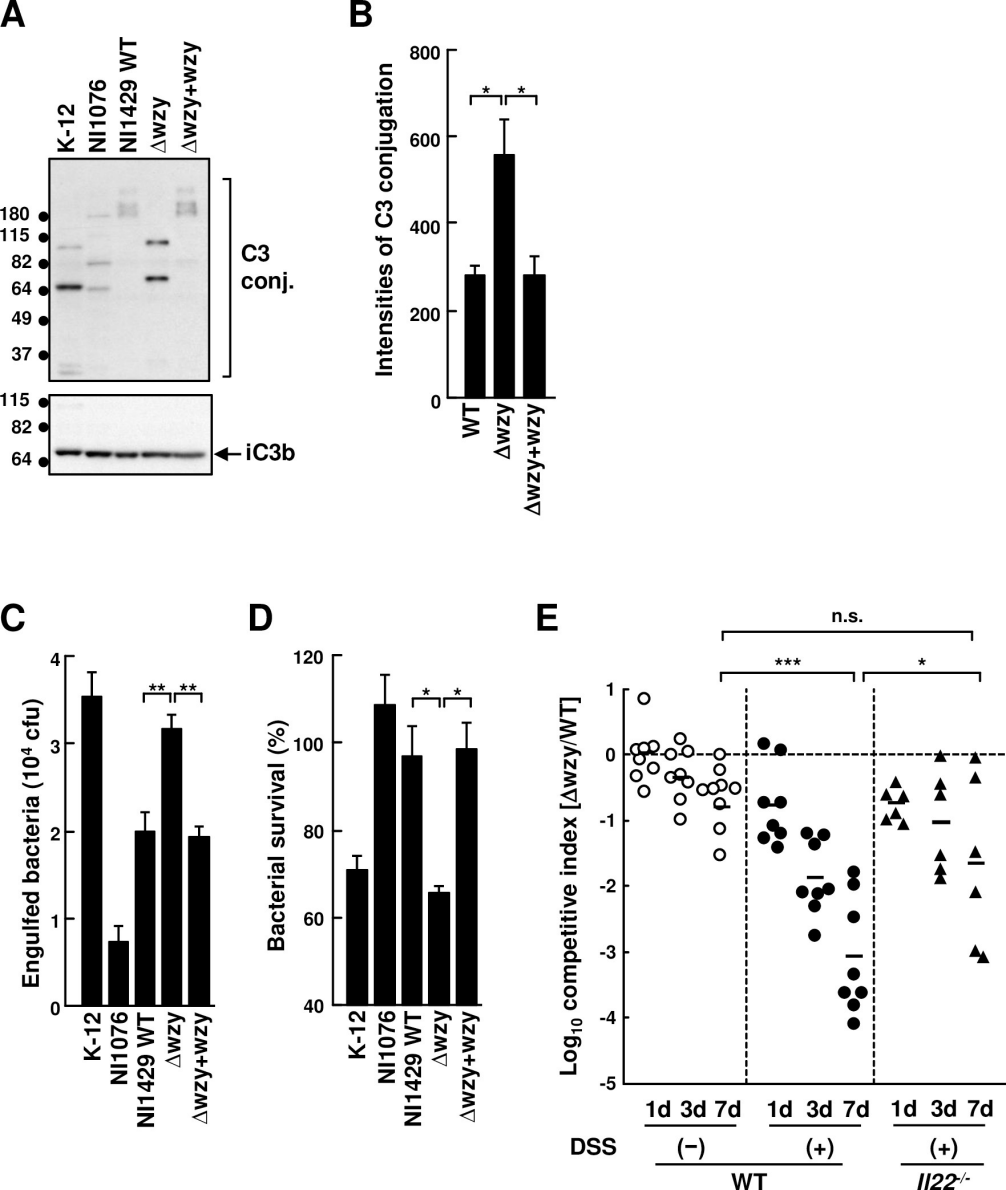
Colitis-associated *E*. *coli* isolates exhibit complement resistance through O antigen structure. (A and B) Indicated bacteria were incubated with fresh mouse serum for 30 min. (A) C3 deposition on bacteria (upper panel) and C3 processing (lower panel) were determined by immunoblotting. (B) Quantification of C3 deposition on indicated bacteria (n = 4). (C) **Bone marrow macrophages were incubated with the indicated bacterial strains in 5% fresh mouse sera. Internalized bacteria were counted after treatment with gentamicin (n = 3). (D) Peritoneal neutrophils were incubated with the indicated strains in 2.5% fresh mouse serum for 2 hr. Surviving bacteria were counted by plating (n = 3). Δ*wzy*; NI1429Str Δ*wzy***, **Δ*wzy*+ *wzy*;** NI1429Str**Δ***wzy*[pGEM-T-w*zy*]. (E) WT and *Il22*^*−/−*^ mice were inoculated with a mixture of equal numbers of NI1429Str (WT) and NI1429StrΔ*wzy*::Cm (Δ*wzy*) bacteria after treatment with streptomycin for 1 day, followed by administration of DSS or mock for 7 days and regular water for 1 day. The numbers of WT and the *wzy* mutant bacteria were determined after the indicated days of DSS treatment and used to measure the competitive index. Competitive index was calculated by logarithmic ratio of number of Δ*wzy* against number of WT at the indicated time points (**WT; n = 8,** *Il22*^*−/−*^; **n = 6**). Data represent pooled results from two independent experiments. Error bars represent SEM. *p < .05, **p < .01.

A genetic feature of disease-associated *E*. *coli* strains is the presence of particular O- and K- serotypes, which reflect differences in the surface polysaccharide layer composed of lipopolysaccharides (LPS) and capsular polysaccharides (CPS), respectively. Both LPS and CPS provide resistance against complement-mediated defense mechanisms [4], and thus colitis-associated *E*. *coli* strains have the potential to be virulent in the host. *In silico* serotyping by Serotypefinder and BLAST showed that the serotypes of colitis-associated *E*. *coli* NI1429 and NI1522 were O54-like:K-:H45, whereas that of NI1413 was O7:K1:H7. To test whether the lipopolysaccharide O structure is important for sensitivity to complement and engulfment by phagocytes, we generated an isogenic mutant strain of NI1429 lacking the surface polysaccharide layer by deletion of the *wzy* gene (Δ*wzy*) by homologous recombination and its plasmid-complemented strain (Δ*wzy* + *wzy*) [30, 31]. Biochemical analysis confirmed that the *wzy* mutant lacked O-antigen of LPS and its complementation with a *wzy* plasmid restored O-antigen expression (**S3 Fig. Panel A**). Furthermore, the *wzy* mutant grew at a comparable rate to the WT strain *in vitro*, and its inhibitory activity against LF82 remained intact (**S3B and S3C Fig**). To assess the C3 binding ability, we compared C3-deposition levels on the *E*. *coli* NI1429 strain after incubation with fresh mouse serum as a source of C3. K-12 and NI1076 were used as complement sensitive and resistant controls, respectively [6]. Consistent with previous studies [32, 33], the *wzy* mutant conjugated more C3 than the parental WT strain and the *wzy* complemented mutant strain (**Fig 3A and 3B**). Furthermore, the *wzy* mutant showed increased engulfment by bone marrow-derived macrophages and susceptibility to killing by peritoneal neutrophils compared with the WT strain (**Fig 3C and 3D**). In addition, the *wzy* mutant showed reduced ability to adhere to and invade intestinal epithelial cells *in vitro* compared with the WT strain (**S3D and S3E Fig**). Collectively, these results indicate that the surface polysaccharide layer, which depends on *wzy*, is critical for resistance to complement and phagocyte killing in colitis-associated *E*. *coli*.

Next, we tested if the *wzy* mutant is sensitive to host elimination during colitis *in vivo*. To this end, we pre-treated the mice with streptomycin for 1 day to deplete the microbiota and then co- inoculated the mice orally with an equal mixture of streptomycin-resistant WT NI1429 and its isogenic Δ*wzy* mutant strain. In the absence of DSS treatment, the fitness of the Δ*wzy* mutant to colonize the gut decreased less than one log in mock-treated mice, compared with the WT strain (**Fig 3E**). In contrast, the colonization fitness of the Δ*wzy* mutant decreased ~ 3 logs compared with the WT strain after 7 days of DSS treatment (**Fig 3E**). Furthermore, the competitive index of the Δ*wzy* mutant improved in *Il22*^*−/−*^ mice after DSS treatment when compared with that observed in DSS-treated WT mice (**Figs 3E and S4A**). Previous studies showed that IL-22 limits the systemic colonization of bacterial symbionts through the regulation of the complement system [6]. We found that IL-22 mRNA levels were increased in the colon of DSS-treated WT mice (S4 Fig **Panel B**). Quantitative RT-PCR and immunoblotting analyses showed increased levels of C3 mRNA in the liver and C3 protein in serum of DSS-treated WT mice (**S4C and S4D Fig**). Consistent with previous studies [6], induction of C3 mRNA in the liver and C3 protein in the serum was largely dependent on IL-22 in DSS-treated mice (**S4C and S4D Fig**). Collectively, these results indicate that the Δ*wzy* mutant AIEC exhibits reduced ability to colonize the intestine under colitic conditions and suggest that IL-22 limits the colonization of the Δ*wzy* mutant in the inflamed intestine.

### The *wzy* AIEC mutant alleviates DSS-induced colitis

**Fig 4:**
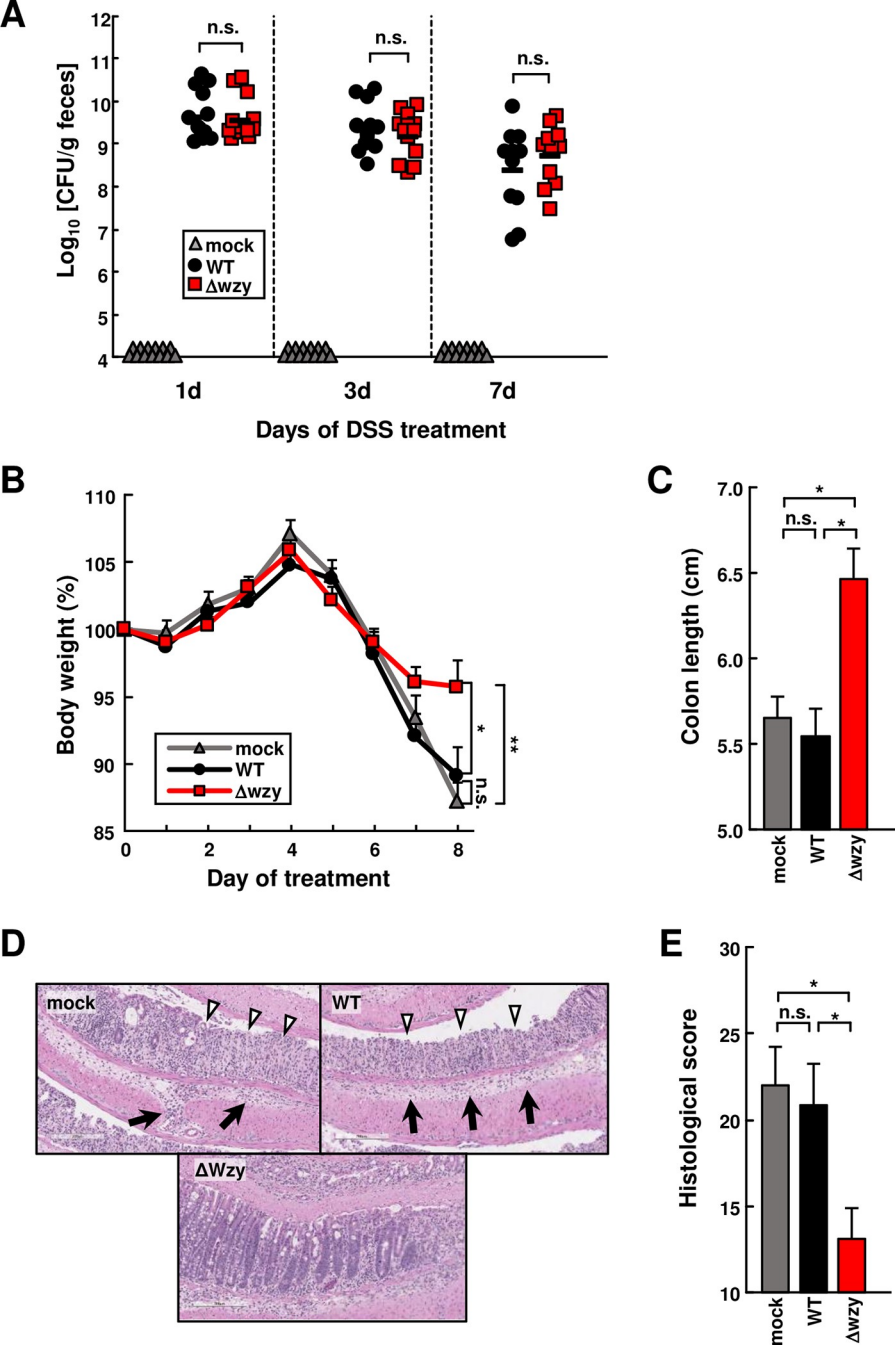
The *wzy* AIEC mutant alleviates DSS-induced colitis. **WT** mice **were inoculated with 1 × 10**^**8**^ **cfu of NI1429Str (WT), NI1429StrΔ*wzy*::Cm (Δwzy) or mock after administration of streptomycin (2mg/ml) in the drinking water for 1 day, followed by DSS treatment for 7 days and regular water for 1 day (n = 13 per group). (A) The numbers of bacteria in feces were determined at the indicated time points. 10**^**4**^ **CFU/g represents the limit of detection using our plating method. (B) Body weight change was monitored over 8 days after treatment with DSS and 1 day of regular water. (C) Colon length. (D and E)** Representative histology of HE–stained colonic sections (D) and histological scores (E) of colons from indicated mice after 7 days of DSS treatment and 1 day of regular water (n = 8 per group). Open arrowheads indicate mucosal ulcer with total loss of the epithelium. Solid arrows indicate presence of submucosal and transmural inflammation and edema. Data represent pooled results from three independent experiments. Error bars represent SEM. *p < .05, **p < .01.

We next assessed whether the Δ*wzy* mutant AIEC is protective against intestinal inflammation. To test this, we pretreated with streptomycin for 1 day and then inoculated the mice orally with streptomycin-resistant WT or Δ*wzy* mutant NI1429 strains in the presence of DSS in the drinking water for 7 days. The colonization levels of the WT strain and the Δ*wzy* mutant strain were comparable after 1, 3 and 7 days of DSS treatment (**Fig 4A**). Furthermore, mice colonized with the WT bacterium and the Δ*wzy* mutant showed comparable weight loss early after DSS treatment, but mice colonized with the Δ*wzy* mutant showed reduced weight loss after 7 days of DSS treatment (**Fig 4B**). The reduced weight loss in mice colonized with the Δ*wzy* mutant was associated with decreased colon shortening and pathology scores when compared with mock-treated or mice colonized with the WT strain (**Figs 4C to 4E, and S5A**). Analysis of bacterial loads showed increased numbers of the WT NI1429 strain compared with the isogenic Δ*wzy* mutant strain in the mesenteric lymph nodes (S5 **Fig** **Panel B**). These results suggest that the lipopolysaccharide O side chain of AIEC regulates intestinal inflammation.

### Host protection against colitis by the *wzy* mutant depends on complement C3

**Fig 5:**
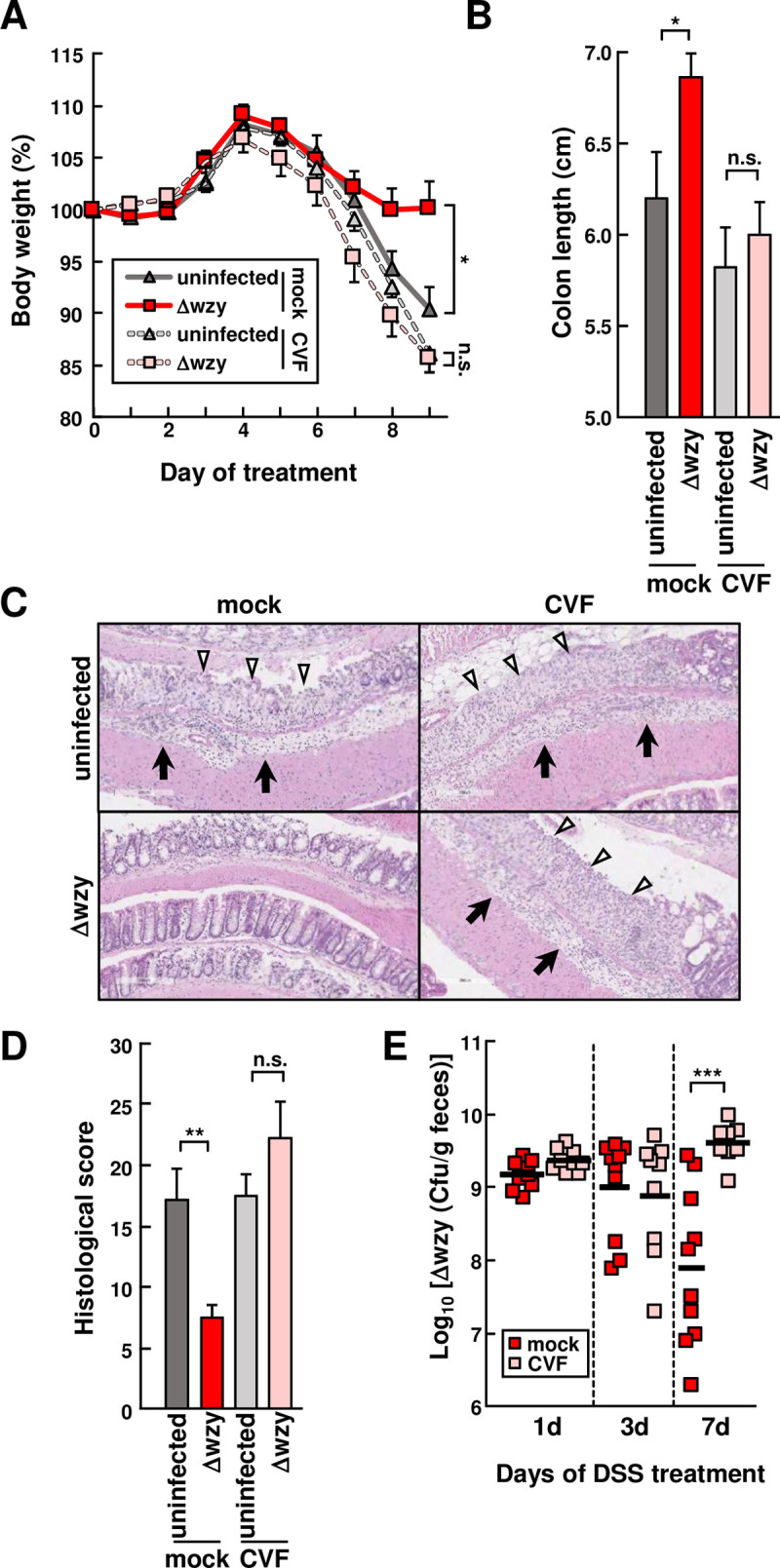
Host protection by the *wzy* AIEC mutant depends on complement C3 WT mice colonized with NI1429StrΔ*wzy*::Cm (Δ*wzy*) were treated intraperitoneally with cobra venom factor (CVF, 25 μg/body) or mock 1 day before and on day 1, 4, 7 after DSS treatment. **Mice were given DSS in the drinking water for 7 days followed by regular water for 2 days (n = 10 per group). (A) Body weight change was monitored over 9 days after treatment with CVF or mock and DSS (B) Colon length. (C and D)** Representative histology of HE–stained colonic sections (C) and histological scores (D) of colons from indicated mice after 7 days of DSS treatment. (E) The number of Δ*wzy* bacteria in feces was determined after the indicated days of DSS treatment. Data represent pooled results from two independent experiments. Error bars represent SEM. *p < .05, **p < .01, ***p < .001.

We first examined whether the protective ability of the *E*. *coli Δwzy* mutant against DSS-induced colitis was associated with changes in the immune populations of the lamina propia in the large intestine. Mice colonized with the *Δwzy* mutant and mock contained similar numbers of monocytes, macrophages, neutrophils and dendritic cells in the intestinal tissue after DSS treatment (**S6 Fig.**). To determine whether reduced colitis associated with colonization by the Δ*wzy* mutant depends on C3, we depleted C3 in WT B6 mice by intraperitoneal administration of cobra venom factor (CVF) [34]. C3 depletion was confirmed by immunoblotting analysis (**S7 Fig. Panel A**), and macrophage engulfment assay that relies on C3 (**S7 Fig. Panel B**). Importantly, treatment with CVF blocked the ability of the Δ*wzy* mutant to protect against DSS-induced colitis as assessed by weight loss, colon length and pathology scores (**Figs 5A to 5D and S7C**). To determine if C3 mediates the protective role of the Δ*wzy* mutant against colitis by regulating bacterial numbers, we assessed the colonization levels of the Δ*wzy* mutant bacterium over time after DSS treatment. After C3 depletion, we found that the loads of the Δ*wzy* mutant were ~ 50-fold higher in mice treated with CVF than in mock-treated mice after 7 days of DSS treatment (**Fig 5E**). In contrast, the intestinal loads of the Δ*wzy* mutant was comparable after 1 and 3 days of DSS treatment in the presence and absence of C3 depletion. Collectively, these results suggest that the anti-inflammatory effect of the Δ*wzy* mutant is dependent on the host complement system.

## Discussion

In this study, we found that *Il22*^*−/−*^ mice exhibit increased susceptibility to DSS-induced colitis in the presence of antibiotic-induced dysbiosis. Treatment of mice with multiple antibiotics induced accumulation of particular bacterial strains belonging to the Enterobacteriaceae family that include AIEC. Such bacteria exhibited unique genetic traits including the presence of polymorphisms of type I fimbriae tip adhesin FimH, genes producing T6SS effectors, genes associated with anti-bacterial immunity such as colicin-containing plasmids and genes for contact-dependent growth inhibition. Unexpectedly, colonization of mice with WT AIEC did not exacerbate DSS-induced colitis. However, an AIEC mutant deficient in *wzy*, a gene that encodes an enzyme responsible for the biosynthesis of the surface polysaccharide layer, conferred protection against colitis. Resistance of bacteria to host defense responses is dependent, at least in part, on structures present on the bacterial surface and in particular O- and K- serotypes expressed by enterobacteria [4]. In pathogenic enterobacteria, these polysaccharide structures are associated with bacterial virulence and fitness at invasion sites [35, 36]. We found that the protective function of surface polysaccharide synthesized by Wzy is primarily observed under inflamed conditions, suggesting a role for host immune factors in the regulation of *wzy*-dependent bacterial fitness. Previous work showed that O- and K- serotypes are important for virulence and resistance to host elimination in ExPEC including uropathogenic and neonatal meningitis-associated *E*. *coli* [37–39]. However, the role of surface polysaccharides of intestinal commensal pathogenic *E*. *coli* (InPEC), including AIEC, in the regulation of intestinal colonization had not been previously investigated. Our studies suggest that the sensitivity of an InPEC strain such as NI1429 to complement is important for regulation of disease activity in the intestine. Consistent with this notion, the loss of surface polysaccharides by the *wzy* deficiency in AIEC alleviated the susceptibility to colitis. Although unlikely, we cannot rule out that mutations potentially acquired during the generation of streptomycin-resistant *E*. *coli* strains could contribute to the protective phenotype in vivo. Nissle 1917, a human *E*. *coli* strain, which has been widely used as a probiotic strain has a *wzy* mutation [32]. Our results suggest that the probiotic activity of Nissle 1917 is, at least in part, explained by the loss of the surface polysaccharide structure which causes susceptibility to complement-mediated elimination. Nissle 1917 produces microcins H47 and M which has been suggested to mediate its ability to outcompete other bacteria and reduce colitis [40, 41]. Therefore, the ability of AIEC strains to outcompete other bacteria via the production of colicins under colitic conditions could be harnessed for the generation of probiotic strains. Because such mutants are expected to occupy the same ecological niche that WT AIEC and related bacteria inhabit, replacement of pro-inflammatory WT bacteria with mutant strains such as those harboring the *wzy* mutation might be beneficial in reducing inflammatory activity. In addition to the role of the interaction between O-antigen and complement system described here, Nissle1917 has been suggested to outcompete other bacteria through various mechanisms [41–44]. Thus, it will be important to determine in future studies the mechanisms by which Nissle1917 and related *E*. *coli* outcompete other bacteria under strong selective pressure conditions such as complement-mediated elimination.

We found that the protective function of the *wzy* mutant against colitis depends on C3. Depletion of C3 resulted in an increased abundance of *E*. *coli* in the intestine of DSS-treated, but not untreated mice, suggesting that C3 controls the number of *E*. *coli* in the intestine only under colitic conditions. Because C3 can be found in the intestinal content [45, 46], the effect of C3 to regulate *E*. *coli* abundance is presumably mediated by C3 released into the intestinal lumen. Several approaches using probiotic bacteria have been proposed for the treatment of IBD [47]. These include the use of bacterial strains such as Nissle 1917 that were selected by functional screens for effectiveness in clinical trials. Nissle 1917 has been used to promote the maintenance of remission in UC patients [48]. Our study demonstrates that the probiotic effect of the *wzy* mutant of colitis-associated *E*. *coli* NI1429 requires host immune factors including C3, suggesting that effective use of probiotic strains based on the *wzy* mutation requires the presence of a normal immune system and in particular an intact complement system. Although the probiotic effect of the Δ*wzy* mutant requires an intact complement system, the precise mechanism is not yet known. One possibility is that complement-sensitive bacteria, which have a high ability to outcompete other bacteria, reduce some pro-inflammatory bacteria and allow the host to eliminate translocated bacteria via C3. Identification of such pro-inflammatory bacteria that are competed out by the Δ*wzy* mutant and further analyses with the Δ*wzy* mutant and other bacteria will reveal the mechanism in which the *wzy*-deficient bacteria works to improve inflammation in the intestine.

## Materials and methods

### Reagents and culture cells

Fc-IL-22 was obtained from AdipoGen LIFE SCIENCE (San Diego, CA). Cobra venom factor (CVF) was purchased from Complement Technology (Tyler, TX). Human intestinal epithelial T84 cells were obtained from ATCC, cultured and maintained with DMEM/F12(1:1) medium (Thermo Fischer Scientific, Waltham, MA) supplemented with 10% fetal bovine serum and 1% penicillin-streptomycin.

### Mice

WT C57BL/6 mice were obtained from the Jackson Laboratory. *Il22*^*−/−*^ mice on C57BL/6 background were a gift from Dr. Wenjun Ouyang (Genentech). All mice were housed, bred, and maintained under specific pathogen-free conditions as described in Hasegawa et al.[6] Mice in different cages were cohoused for 3 weeks for normalization of the microbiota before the experiments. The mouse studies were approved by the University of Michigan Committee on Use and Care of Animals (approved protocol # PRO00008086).

### Bacterial strains, isolation, culture, and mutagenesis of bacteria

All strains and plasmids used in this study are listed in S3 Table. Fecal bacteria were isolated by plating on non-selective bovine heart infusion medium at 37°C under aerobic conditions, further selected by plating on MacConkey medium, and stored with 25% glycerol at -40°C until experiments. Bacterial species were determined by sequencing 16S rRNA genes as described [6]. *E*. *coli* K-12 substr. MG1655 and *Yersinia enterocolitica enterocolitica* ATCC 27729 were obtained from ATCC. *E*. *coli* LF82, AIEC75 and EC93, and *Citrobacter rodentium* DBS120 are gifts of Drs. Tonyia Eaves-Pyles (University of Texas), Kenneth Simpson (Cornell University), Christopher Heynes (University of California), and David Schauer (Massachusetts Institute of Technology), respectively. For experiments, all bacteria were thawed and grown in Miller Luria-Bertani (LB) medium at 37°C under aerobic conditions.

Spontaneous streptomycin(Str)-resistant strains were generated by successive culture of bacteria on BHI medium containing increasing concentrations (25, 50, 200, 1000, 2000 ug/ml) of streptomycin as described[49]. After cloning candidates, we verified that their growth rates and maximal growth densities were similar to those of the parental strains. Targeted deletion of *fimH* and *wzy* of NI1429 was performed by homologous recombination using the pKD46 system as described [50]. Briefly, NI1429 was transformed with pKD46 and then the chromosomal genes were replaced by a chloramphenicol resistance cassette from the plasmid pKD3 plasmid using a PCR fragment amplified with primers shown in S4 Table. The chloramphenicol (Cm) resistance cassette was removed using the pCP20 plasmid except for NI1429strΔ*wzy*::Cm. The deletion of the target gene and the removal of Cm gene was confirmed by PCR using primers listed in S4 Table. For complementation, the full open reading frame (ORF) of the gene was amplified from the genomic DNA of NI1429 parent strain by PCR using primers shown in S4 Table. The ORF was inserted into pGEM-T Easy by commercial TA-cloning kit (Promega, Madison, WI), followed by the transformation into the *wzy* mutants. The endogenous plasmid pNI1429 was isolated from NI1429 using a commercial kit (Qiagen, Hilden, Germany). The ampicillin gene was amplified from pGEM-T Easy by using Amp5Bam and Amp3XhoI and inserted into BamHI and XhoI sites of pNI1429 to generate pNI1429Amp. The mutant plasmid pNI1429AmpΔCol lacking the region containing *colY* gene, was generated by the removal of EcoRI fragments in the *colY* gene. The constructs pNI1429Amp and pNI1429AmpΔCol were confirmed using primer sets shown in S4 Table. The strains carrying the resulted plasmids were generated by transformation of the NI1429Str with the plasmids and successive culture on L-broth plates containing 100 μg/ml ampicillin until loss of the endogenous pNI1429 and monitoring with Y5Bam/Y3XbaI. The authenticity of all plasmids was verified by DNA sequencing.

### Genome sequencing and microbiota composition analyses

For genomic sequencing, genomic DNA of bacteria was isolated as described [51] and subjected to random sequencing by Illumina MiSeq. The resulted paired-end sequences were assembled to contigs by Velvet [52], and genes were annotated by Prokka [53] after quality check. Ortholog gene groups and the phylogenetic tree distances of *E*. *coli* strains were determined by roary[54], after reannotating reference genomes, which are available in GenBank, by Prokka. For microbiota composition analysis, bacterial DNA was extracted from mouse feces using QIAamp DNA Stool Mini Kit (QIAGEN, Hilden, Germany) as instructed by the manufacturer. The V4 region of the 16S rRNA gene (252 bp) was sequenced with an Illumina MiSeq, and analyzed by using Mothur[55, 56]. OTUs were classified into taxons at >97% identity with the use of Mothur[55]. We also used OTUs at 100% identity to determine detailed bacterial taxons by BLASTN. Spearman ranking analysis was performed using otu.association of Mothur after adding normalization for Lcn2 level and OTU abundance. Bacterial serotypes were determined using genomic sequences and SerotypeFinder (https://cge.cbs.dtu.dk/services/serotypefinder/). Phylogroups of the isolates were determined by the presence of ChuA, yjaA and TSPE4.C2 [57]. To determine the most associated co-abundance group (CAGs) with Lcn2 levels, logarithmic values of OTU abundance and Lcn2 level were clustered with MeV (http://www.tm4.org/) by a complete linkage method using Pearson's squared correlation coefficients. The distant tree was visualized by iTol (https://itol.embl.de/).

### Adhesion and invasion assays

Adhesion and invasion assays were performed as previously described [28]. Briefly, bacteria were cultured in L-broth medium at 37°C for 18 hours under aerobic conditions. human intestinal epithelial T84 cells were cultured for 14 days with DMEM/F12(1:1) to polarize the cells. Polarized T84 cells were infected with the indicated strains at a multiplicity of infection (MOI) of 10 for 3 hr. For adhesion assays, cells were lysed with 0.1% Nonidet P-40 after washed with PBS and the number of bacteria associated with cells was determined by plating on L-broth. For invasion assays, cells were washed with PBS, followed by treatment with 100 μg/ml of gentamicin for 1 hr to kill extracellular bacteria. Then, cells were lysed with 0.1% Nonidet P-40 and the number of internalized bacteria was determined by plating.

### *In vitro* competition assay

For halo assays, the L-Broth agar plates were overlaid with the target bacteria strain onto 0.5% agar. Then, the formation of an inhibition zone was evaluated by spotting 3 μl of serial 1:10 dilutions of each competitor strain (starting at OD_600_ = 1.0) followed by incubation of the plates at 37°C overnight. For growth inhibition of LF82, LF82 and indicated strains of NI1429 were cultured at a starting OD_600_ of 0.05. After 3hr, bacteria numbers of LF82 and NI1429 strains were determined by plating on L-broth agar supplemented with 100 μg/ml ampicillin and 200 μg/ml of streptomycin, respectively.

### Structural analysis of lipopolysaccharides (LPS)

LPS was prepared by phenol extraction method as described [58]. A total of 2 x10^9^ bacteria were suspended with lysis buffer (100 mM SDS, 50 mM Tris-Cl, 0.128 M NaCl) and digested with 0.1mg/ml protease K at 56°C for 1 hr. An equal volume of TE-saturated phenol/chloroform/isoamyl alcohol (25:24:1,v/v) was added, then heated at 65°C for 15 min. After centrifugation, LPS was precipitated by adding two volumes of ethanol to the aqueous phase and resuspended in distilled water. After electrophoresis using a 12% SDS-PAGE gel, LPS was detected with Silver Staining (Thermo Fisher Scientific).

### Immunoblotting analysis

The serum levels, deposition of C3 on bacteria, and C3 processing were determined by immunoblotting using anti-C3d polyclonal antibody as described.[6] Briefly for C3 deposition assay, a total of 5 × 10^8^ cfu of bacteria were incubated with 10% (v/v) indicated mouse serum for 30 min at 37°C. Unbound C3 was removed by extensive washing with ice cold PBS. Samples were boiled in Laemmli's buffer and separated by 10% SDS-PAGE (1 × 10^8^ per lane). 1/100 volume of the reaction mixture was used as total protein control.

### Macrophage phagocytosis and neutrophil bactericidal assays

Macrophage phagocytosis and neutrophil bactericidal assays were performed as described[6]. Briefly, macrophages were derived from bone marrow as described [59]. Macrophages were infected with the indicated bacterial strains at an MOI of 1 for 20 min with 5% fresh mouse serum and treated with 100 μg/ml of gentamicin for 1 hr before being lysed with 0.1% Nonidet P-40. The number of internalized bacteria was determined by plating. For neutrophil bactericidal assay, mouse peritoneal neutrophils were collected from the abdominal cavity of mice 6 hr after intraperitoneal injection with thioglycollate broth as described [6]. A total of 5 × 10^5^ neutrophils were incubated with 5 × 10^3^ bacteria with 5% fresh mouse serum for 2 hr. The number of survival bacterial was determined by plating.

### DSS-induced colitis

Mice (8 to 10-week old females) were co-housed for microbiota normalization for 2–3 weeks before treatment with 2.5 (w/v) % DSS (molecular weight 40,000–50,000; Affymetrix, Santa Clara, CA) in the drinking water for 7 days, followed by regular water for 1 day. For induction of dysbiosis, WT and *Il22*^*−/−*^ mice were pretreated with an antibiotic (Abx) cocktail (ampicillin, kanamycin, gentamicin, colistin, metronidazole, and vancomycin) in the drinking water for 6 days and intraperitoneally injected with clindamycin one day before DSS treatment as described [60]. For colonization of *E*. *coli* strains, WT and *Il22*^*−/−*^ mice were pretreated with streptomycin (2 mg/ml) in the drinking water for 1 day, then inoculated orally with 1 × 10^8^ colony-forming units (cfu) of streptomycin-resistant *E*. *coli* before DSS treatment. For depletion of complement C3, mice **were treated intraperitoneally with cobra venom factor (25 μg/body) or mock 1 day before and on day 1, 4, 7 after DSS treatment.** Disease was monitored daily by measuring body weight and by collecting fecal pellets for the measurement of lipocalin 2 (Lcn2) levels and bacterial loads. On day 7, DSS was removed from drinking water and 48 hr later mice were euthanized and organs were removed for mRNA analysis and histological analysis. Blood was collected eight days after DSS treatment. Histological scores were evaluated as the sum of three parameter as follows; severity of inflammation (0, none; 1, mild; 2, moderate; 3, severe), the level of involvement (0, none; 1, mucosa, 2; mucosa and submucosa; 3, transmural), and extent of epithelial/crypt damage (0, none; 1, basal 1/3; 2, basal 2/3; 3, crypt loss; 4, crypt and surface epithelial destruction). Each score was multiplied by a factor of 1–4 [1, 0–25%; 2, 26–50%; 3, 51–75%; 4, 76–100%] according to the percentage of the colon involved [61]. Bacterial loads in feces were determined by plating on MacConkey agar plates supplemented with 200 μg/ml streptomycin. Fecal lipocalin 2 levels were determined using a commercial kit (Mouse Lipocalin 2 DuoSet ELISA, R&D Systems, Minneapolis, MN) as described[26].

### mRNA quantification

Relative expression levels of gene mRNA in colon or liver were determined by quantitative RT-PCR. Briefly, colonic tissues or liver were homogenized in Trizol (Thermo Fisher Scientific) and RNA was purified using PureLink RNA Mini Kit (Thermo Fisher Scientific). cDNA was synthesized using SuperScript II Reverse Transcriptase (Thermo Fisher Scientific) and real-time PCR was performed using ABI 7900HT system (Thermo Fisher Scientific). The PCR primers used in this study are listed in S4 Table. Values were normalized to *gapdh* levels.

### Intestinal cell isolation and flow cytometric analysis

Hematopoietic cells were isolated from colonic lamina propria, as previously described [62]. Flow cytometric analysis was performed using BD FACSCelesta (Becton Dickinson Biosciences, Franklin Lakes, NJ) and data were analyzed using FlowJo software (Becton Dickinson Biosciences). Fluorescence-conjugated antibodies against CD11b (M1/70), CD11c (N418), MHC class II (M5/114.15.2), Ly6C (HK1.4), Ly6G (RB6-8C5), and CD45 (30F-11), were purchased from eBioscience (San Diego, CA). Neutrophil, macrophage, monocyte, dendritic cell populations were defined by the presence of surface makers Ly6G^+^MHCII^high^CD11b^+^CD45^+^, MHCII^high^Ly6C^+^CD11b^+^CD45^+^, MHCII^low^Ly6C^+^CD11b^+^CD45^+^, and CD11c^+^CD11b^-^CD45^+^, respectively. The numbers of individual cell types were calculated from the percentages of detected cell types and total hematopoietic cell numbers.

### Statistical analysis

Statistical analyses were performed using GraphPad Prism software version 8 (GraphPad Software Inc.). Difference between two groups was evaluated with two-tailed Student's t test. For multiple group comparisons, statistical analysis was performed with one-way ANOVA, followed by Tukey-Kramer's or Dunnett's post hoc test. Differences at P < .05 were considered significant.

## Supporting information

S1 Fig Detailed histological scores and abundance of Enterobacteriaceae by qPCR shown in Fig 1. (A) Individual parameters of histological scores shown in Fig 1E. (B) Abundance of Enterobacteriaceae quantified by qPCR shown in Fig 1A. Values were normalized to the mean of day 0.
(PDF)

S2 Fig Phylogenetic distances, virulence genes, and competition assay of E. coli strains. (A) Phylogenetic tree of colitis-associated E. coli and representative non-AIEC strains. Phylogenetic distance was calculated as indicated in Methods. Colitis-associated E. coli isolates used in this study are highlighted in yellow. (B) AIEC-related putative virulence genes found in the genomes of E. coli isolates NI1413, NI1429, NI1522 and reference AIEC strains. Red denotes the presence while black indicates the absence of the gene. (C) Representative images of halo assay performed on the lawn of K-12 target cells. The tested competitors were NI1413 and its derivative NI1413Str, NI1396, another strain isolated from colitic Il22-/- mice, isolates from C. difficileinfected Il22-/- mice (NI1165, NI1163, NI1159, NI1153, NI1090, [6]), NI491 isolated from feces of normal mice [6], and its derivative NI491Str, K-12 and its derivative DH5α. The details of the halo assay are described in Materials and Methods.
(PDF)

S3 Fig LPS structure, growth rate, inhibitory effect on growth of LF82, and adhesion and invasion activity of WT and Δwzy E. coli. strains. (A) Extracted LPS from indicated bacteria was visualized with silver staining. (B) Bacteria (optical density (OD), 0.01) was grown in L-broth, and OD was continuously monitored. K-12; K-12 substr. MG1655, Δwzy; NI1429StrΔwzy::Cm, Δwzy+wzy; NI1429StrΔwzy[pGEM-T-wzy]. (C) LF82 was cultured alone or co-cultured with indicated bacteria at 1:1 for 3 hr, then the percentage of cocultured LF82 bacteria compared with LF82 cultured alone was calculated. (D and E) T84 intestinal epithelial cells were infected with indicated bacteria at a MOI of 10. The number of adhered bacteria per cell (D) and the percentage of internalized bacteria compared with the number of initial bacteria (E) are shown. WT; NI1429Str, Δwzy; NI1429StrΔwzy, Δwzy+wzy; NI1429StrΔwzy[pGEM-T-wzy]. Error bars represent SEM. *p < .05, **p < .01.
(PDF)

S4 Fig IL-22-dependent augmentation of C3 deposition on colitis-associated E. coli. (A) WT and Il22−/−mice were inoculated with a mixture of equal numbers of NI1429Str (WT) and NI1429StrΔwzy::Cm (Δwzy) bacteria after treatment with streptomycin for 1 day, followed by administration of DSS or mock for 7 days and regular water for 1 day. The numbers of WT and the wzy mutant bacteria were determined by plating at the indicated time points. The results from this experiment correspond to those shown in Fig 3E. (B) Relative expression levels of Il22 mRNA in the colon of WT and Il22−/− mice treated with or without DSS. N. D., not detected. (C) mRNA expression levels of C3 in the liver of WT mice, DSS-treated WT mice, and DSS-treated Il22−/− mice. (n = 5–6) (D) C3 levels in serum of WT mice, DSS-treated WT mice, and DSS-treated Il22−/− mice. WT; NI1429Str, Δwzy; NI1429StrΔwzy. Error bars represent SEM. *p < .05, **p < .01.
(PDF)

S5 Fig Decreased bacterial loads of the wzy AIEC mutant in the mesenteric lymph nodes after DSS treatment. (A) Individual parameters of histological scores shown in Fig 4E. (B)The numbers of tested bacteria in mesenteric lymph node (MLN) and liver after 7 days of DSS treatment and 1 day of regular water. Error bars represent SEM. *p < .05.
(PDF)

S6 Fig Similar numbers of macrophages, neutrophils and dendritic cells in the intestinal tissue of WT- and Δwzy-colonized mice after 7 days of DSS treatment. Absolute numbers of macrophages (CD45+ CD11b+ MHC II+ Ly6C-), monocytes (CD45+ CD11b+ LY6C+ MHC II-), DCs (CD45+ CD11c+ MHC II+) and neutrophils (CD45+ CD11b+ Ly6G+) in the colon (n = 4–5).
(PDF)

S7 Fig C3 depletion and engulfment activity after treatment with CVF, and detailed histological scores shown in Fig 5. (A) Sera were collected from WT mice treated intraperitoneally with 25 μg/body of cobra venom factor or mock. Serum C3 was determined by immunoblotting. (B) Bone marrow macrophages were incubated with the indicated bacterial strains with or without 5% sera from WT mice treated with 25 μg/body of cobra venom factor or mock. Internalized bacteria were counted after treatment with gentamicin (n = 3). (C) Individual parameters of histological scores shown in Fig 5C. Data represent pooled results from two independent experiments. Error bars represent SEM. ***p < .001.
(PDF)

S1 Table Taxonomic microbiota composition and lipocalin-2 (Lcn-2) levels in feces of representative mice on day 0, 3 and 7 after DSS treatment. (XLSX)

S2 Table Gene orthlogue groups found in E. coli strains. (DOCX)

S3 Table E. coli strains and plasmids used in this study. (DOCX)

S4 Table PCR primers used in this study. (DOCX)
