# Supplementary material for: Lipopolysaccharide O structure of adherent and invasive Escherichia coli regulates intestinal inflammation via complement C3
Source: PLoS Pathog. 2020 Oct 7;16(10):e1008928. doi: 10.1371/journal.ppat.1008928 (PMC7571687; doi:10.1371/journal.ppat.1008928)

S1 Fig. Detailed histological scores and abundance of Enterobacteriaceae by qPCR shown in Fig. 1.

(A) Individual parameters of histological scores shown in Fig. 1E. (B) Abundance of Enterobacteriaceae quantified by qPCR shown in Fig. 1A. Values were normalized to the mean of day 0.

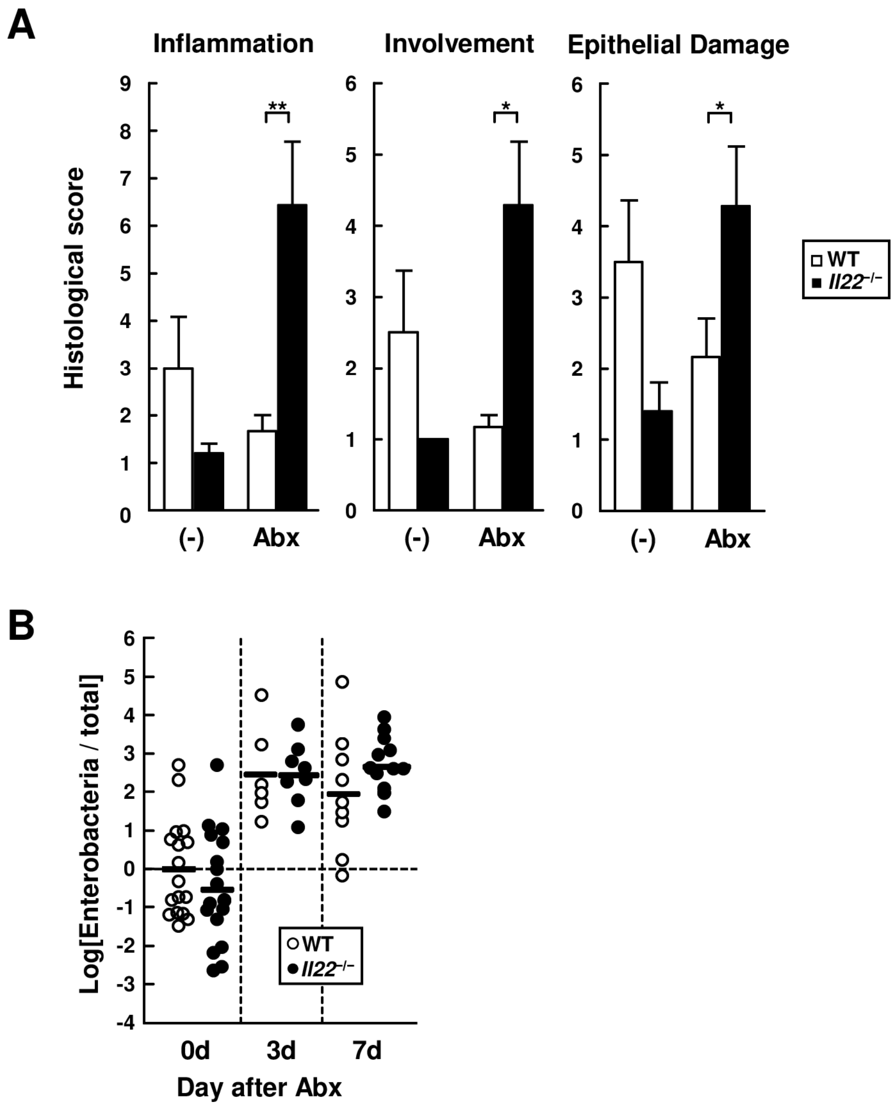

Supplement: S1 Fig — (A) Individual parameters of histological scores shown in Fig 1E. (B) Abundance of Enterobacteriaceae quantified by qPCR shown in Fig 1A. Values were normalized to the mean of day 0. (PDF) [file ppat.1008928.s001.pdf]
