# Supplementary material for: Lipopolysaccharide O structure of adherent and invasive Escherichia coli regulates intestinal inflammation via complement C3
Source: PLoS Pathog. 2020 Oct 7;16(10):e1008928. doi: 10.1371/journal.ppat.1008928 (PMC7571687; doi:10.1371/journal.ppat.1008928)
Supplement: S2 Fig — (A) Phylogenetic tree of colitis-associated E. coli and representative non-AIEC strains. Phylogenetic distance was calculated as indicated in Methods. Colitis-associated E. coli isolates used in this study are highlighted in yellow. (B) AIEC-related putative virulence genes found in the genomes of E. coli isolates NI1413, NI1429, NI1522 and reference AIEC strains. Red denotes the presence while black indicates the absence of the gene. (C) Representative images of halo assay performed on the lawn of K-12 target cells. The tested competitors were NI1413 and its derivative NI1413Str, NI1396, another strain isolated from colitic Il22-/- mice, isolates from C. difficileinfected Il22-/- mice (NI1165, NI1163, NI1159, NI1153, NI1090, [6]), NI491 isolated from feces of normal mice [6], and its derivative NI491Str, K-12 and its derivative DH5α. The details of the halo assay are described in Materials and Methods. (PDF) [file ppat.1008928.s002.pdf]

S2 Fig. Phylogenetic distances, virulence genes, and competition assay of *E. coli* strains.

(A) Phylogenetic tree of colitis-associated *E. coli* and representative non-AIEC strains. Phylogenetic distance was calculated as indicated in Methods. Colitis-associated *E. coli* isolates used in this study are highlighted in yellow. (B) AIEC-related putative virulence genes found in the genomes of *E. coli* isolates NI1413, NI1429, NI1522 and reference AIEC strains. Red denotes the presence while black indicates the absence of the gene. (C) Representative images of halo assay performed on the lawn of K-12 target cells. The tested competitors were NI1413 and its derivative NI1413Str, NI1396, another strain isolated from colitic *Il22*$^{-/-}$ mice, isolates from *C. difficile*-infected *Il22*$^{-/-}$ mice (NI1165, NI1163, NI1159, NI1153, NI1090, [6]), NI491 isolated from feces of normal mice [6], and its derivative NI491Str, K-12 and its derivative DH5α. The details of the halo assay are described in Materials and Methods.

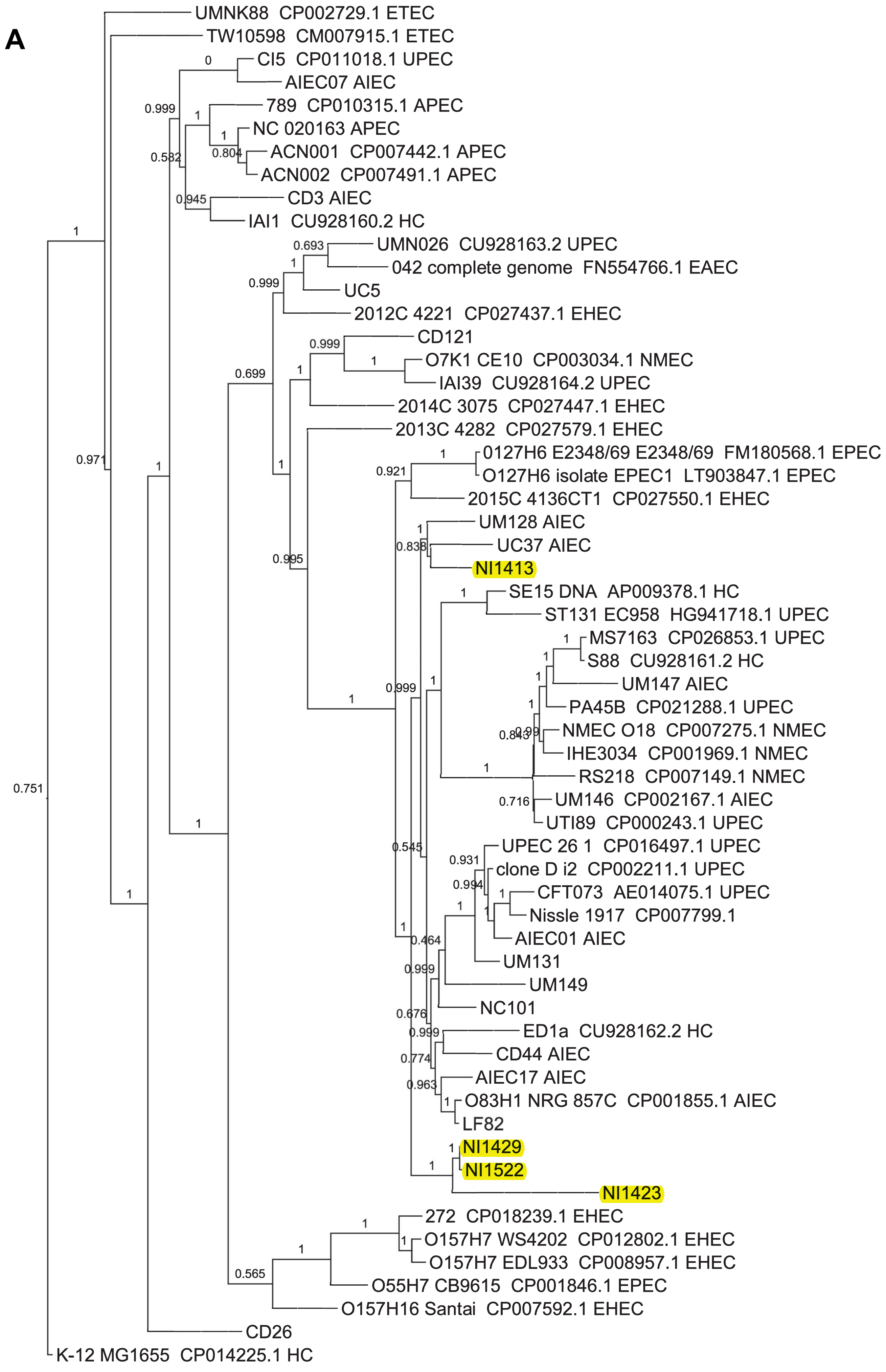
A
UMNK88 CP002729.1 ETEC
TW10598 CM007915.1 ETEC
CI5 CP011018.1 UPEC
AIEC07 AIEC
789 CP010315.1 APEC
NC 020163 APEC
ACN001 CP007442.1 APEC
ACN002 CP007491.1 APEC
CD3 AIEC
IAI1 CU928160.2 HC
UMN026 CU928163.2 UPEC
042 complete genome FN554766.1 EAEC
UC5
2012C 4221 CP027437.1 EHEC
CD121
O7K1 CE10 CP003034.1 NMEC
IAI39 CU928164.2 UPEC
2014C 3075 CP027447.1 EHEC
2013C 4282 CP027579.1 EHEC
0127H6 E2348/69 E2348/69 FM180568.1 EPEC
O127H6 isolate EPEC1 LT903847.1 EPEC
2015C 4136CT1 CP027550.1 EHEC
UM128 AIEC
UC37 AIEC
NI1413
SE15 DNA AP009378.1 HC
ST131 EC958 HG941718.1 UPEC
MS7163 CP026853.1 UPEC
S88 CU928161.2 HC
UM147 AIEC
PA45B CP021288.1 UPEC
NMEC O18 CP007275.1 NMEC
IHE3034 CP001969.1 NMEC
RS218 CP007149.1 NMEC
UM146 CP002167.1 AIEC
UTI89 CP000243.1 UPEC
UPEC 26 1 CP016497.1 UPEC
clone D i2 CP002211.1 UPEC
CFT073 AE014075.1 UPEC
Nissle 1917 CP007799.1
AIEC01 AIEC
UM131
UM149
NC101
ED1a CU928162.2 HC
CD44 AIEC
AIEC17 AIEC
O83H1 NRG 857C CP001855.1 AIEC
LF82
NI1429
NI1522
NI1423
272 CP018239.1 EHEC
O157H7 WS4202 CP012802.1 EHEC
O157H7 EDL933 CP008957.1 EHEC
O55H7 CB9615 CP001846.1 EPEC
O157H16 Santai CP007592.1 EHEC
CD26
K-12 MG1655 CP014225.1 HC

B

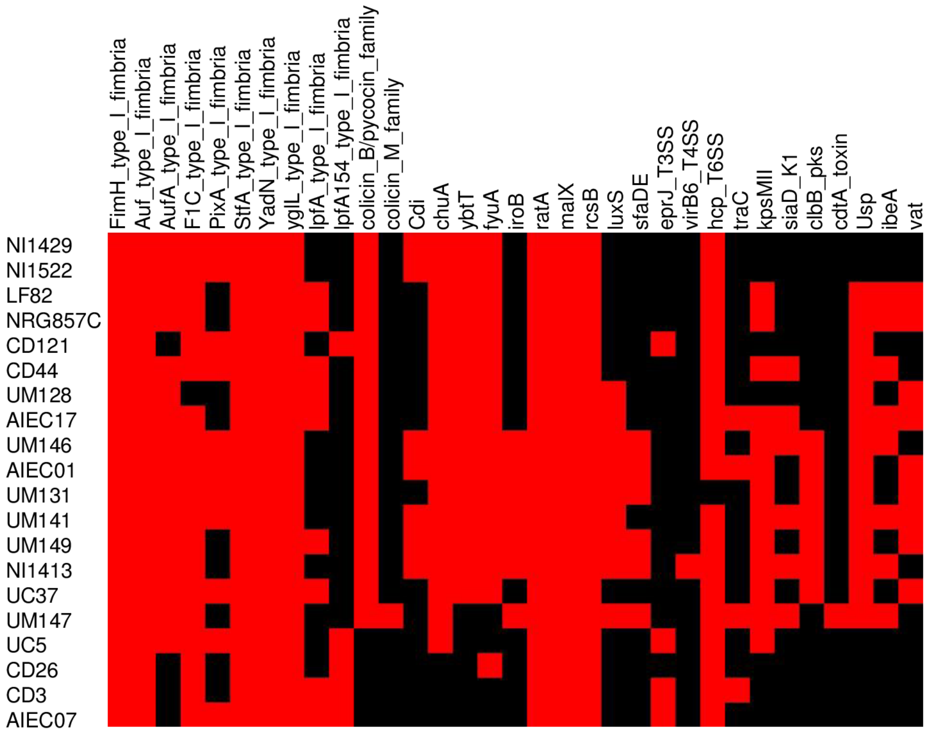
FimH_type_I_fimbria
Auf_type_I_fimbria
AufA_type_I_fimbria
F1C_type_I_fimbria
PixA_type_I_fimbria
StfA_type_I_fimbria
YadN_type_I_fimbria
yglL_type_I_fimbria
lpfA_type_I_fimbria
lpfA154_type_I_fimbria
colicin_B/pycocin_family
colicin_M_family
Cdi
chuA
ybtT
fyuA
iroB
ratA
malX
rcsB
luxS
sfaDE
eprJ_T3SS
virB6_T4SS
hcp_T6SS
traC
kpsMII
siaD_K1
clbB_pks
cdtA_toxin
Usp
ibeA
vat
NI1429
NI1522
LF82
NRG857C
CD121
CD44
UM128
AIEC17
UM146
AIEC01
UM131
UM141
UM149
NI1413
UC37
UM147
UC5
CD26
CD3
AIEC07

C

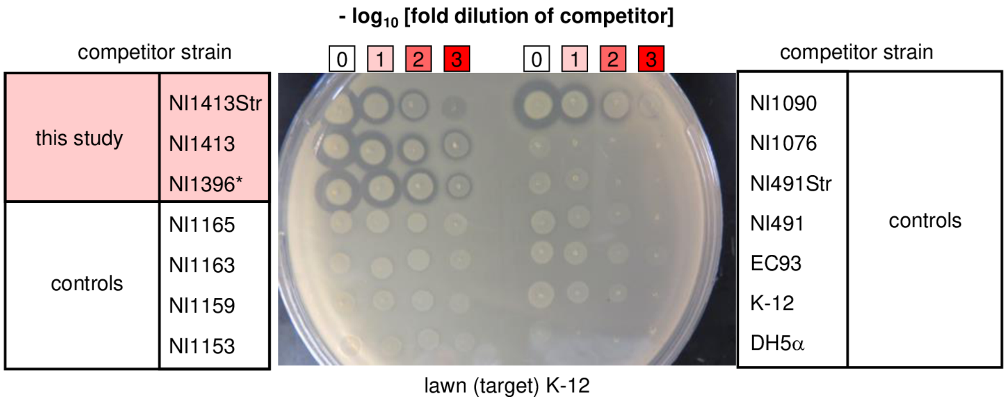
- log10 [fold dilution of competitor]
competitor strain
0
1
2
3
0
1
2
3
competitor strain
this study
NI1413Str
NI1413
NI1396*
controls
NI1165
NI1163
NI1159
NI1153
NI1090
NI1076
NI491Str
NI491
EC93
K-12
DH5α
controls
lawn (target) K-12
