# Supplementary material for: Lipopolysaccharide O structure of adherent and invasive Escherichia coli regulates intestinal inflammation via complement C3
Source: PLoS Pathog. 2020 Oct 7;16(10):e1008928. doi: 10.1371/journal.ppat.1008928 (PMC7571687; doi:10.1371/journal.ppat.1008928)

S3 Fig. LPS structure, growth rate, inhibitory effect on growth of LF82, and adhesion and invasion activity of WT and Δ*wzy* *E. coli.* strains.

(A) Extracted LPS from indicated bacteria was visualized with silver staining. (B) Bacteria (optical density (OD), 0.01) was grown in L-broth, and OD was continuously monitored. K-12; K-12 substr. MG1655, Δ*wzy*; NI1429StrΔ*wzy*::Cm, Δ*wzy*+*wzy*; NI1429StrΔ*wzy*[pGEM-T-w*zy*]. (C) LF82 was cultured alone or co-cultured with indicated bacteria at 1:1 for 3 hr, then the percentage of co-cultured LF82 bacteria compared with LF82 cultured alone was calculated. (D and E) T84 intestinal epithelial cells were infected with indicated bacteria at a MOI of 10. The number of adhered bacteria per cell (D) and the percentage of internalized bacteria compared with the number of initial bacteria (E) are shown. WT; NI1429Str, Δ*wzy*; NI1429StrΔ*wzy*, Δ*wzy*+*wzy*; NI1429StrΔ*wzy*[pGEM-T-*wzy*]. Error bars represent SEM. *$p < .05$, **$p < .01$.

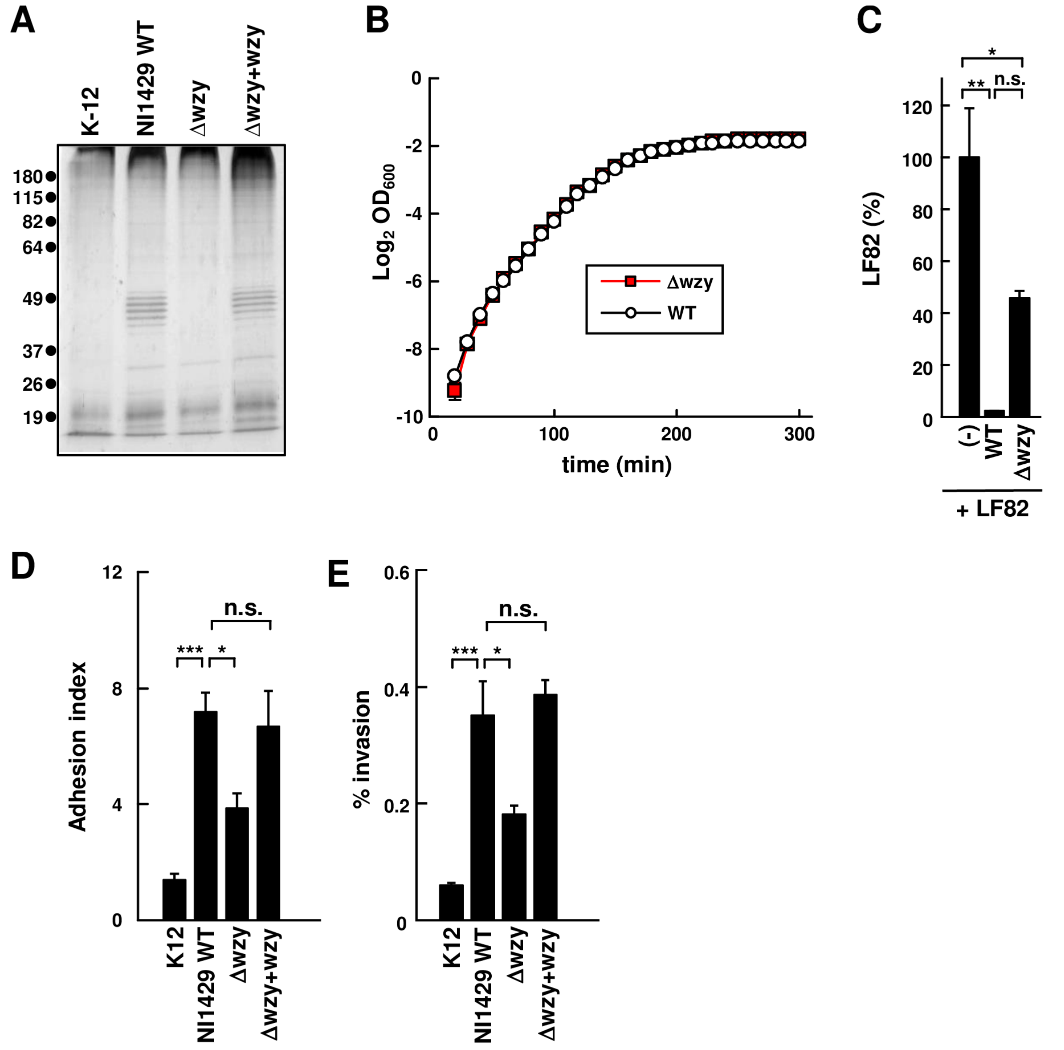

Supplement: S3 Fig — (A) Extracted LPS from indicated bacteria was visualized with silver staining. (B) Bacteria (optical density (OD), 0.01) was grown in L-broth, and OD was continuously monitored. K-12; K-12 substr. MG1655, Δwzy; NI1429StrΔwzy::Cm, Δwzy+wzy; NI1429StrΔwzy[pGEM-T-wzy]. (C) LF82 was cultured alone or co-cultured with indicated bacteria at 1:1 for 3 hr, then the percentage of cocultured LF82 bacteria compared with LF82 cultured alone was calculated. (D and E) T84 intestinal epithelial cells were infected with indicated bacteria at a MOI of 10. The number of adhered bacteria per cell (D) and the percentage of internalized bacteria compared with the number of initial bacteria (E) are shown. WT; NI1429Str, Δwzy; NI1429StrΔwzy, Δwzy+wzy; NI1429StrΔwzy[pGEM-T-wzy]. Error bars represent SEM. *p < .05, **p < .01. (PDF) [file ppat.1008928.s003.pdf]
