# Supplementary material for: Lipopolysaccharide O structure of adherent and invasive Escherichia coli regulates intestinal inflammation via complement C3
Source: PLoS Pathog. 2020 Oct 7;16(10):e1008928. doi: 10.1371/journal.ppat.1008928 (PMC7571687; doi:10.1371/journal.ppat.1008928)

S4 Fig. IL-22-dependent augmentation of C3 deposition on colitis-associated *E. coli*.

(A) WT and *Il22*$^{-/-}$ mice were inoculated with a mixture of equal numbers of NI1429Str (WT) and NI1429StrΔ*wzy*::Cm (Δ*wzy*) bacteria after treatment with streptomycin for 1 day, followed by administration of DSS or mock for 7 days and regular water for 1 day. The numbers of WT and the *wzy* mutant bacteria were determined by plating at the indicated time points. The results from this experiment correspond to those shown in Fig. 3E. (B) Relative expression levels of *Il22* mRNA in the colon of WT and *Il22*$^{-/-}$ mice treated with or without DSS. N. D., not detected. (C) mRNA expression levels of *C3* in the liver of WT mice, DSS-treated WT mice, and DSS-treated *Il22*$^{-/-}$ mice. (n = 5-6) (D) C3 levels in serum of WT mice, DSS-treated WT mice, and DSS-treated *Il22*$^{-/-}$ mice. WT; NI1429Str, Δ*wzy*; NI1429StrΔ*wzy*. Error bars represent SEM. **p* < .05, ***p* < .01.

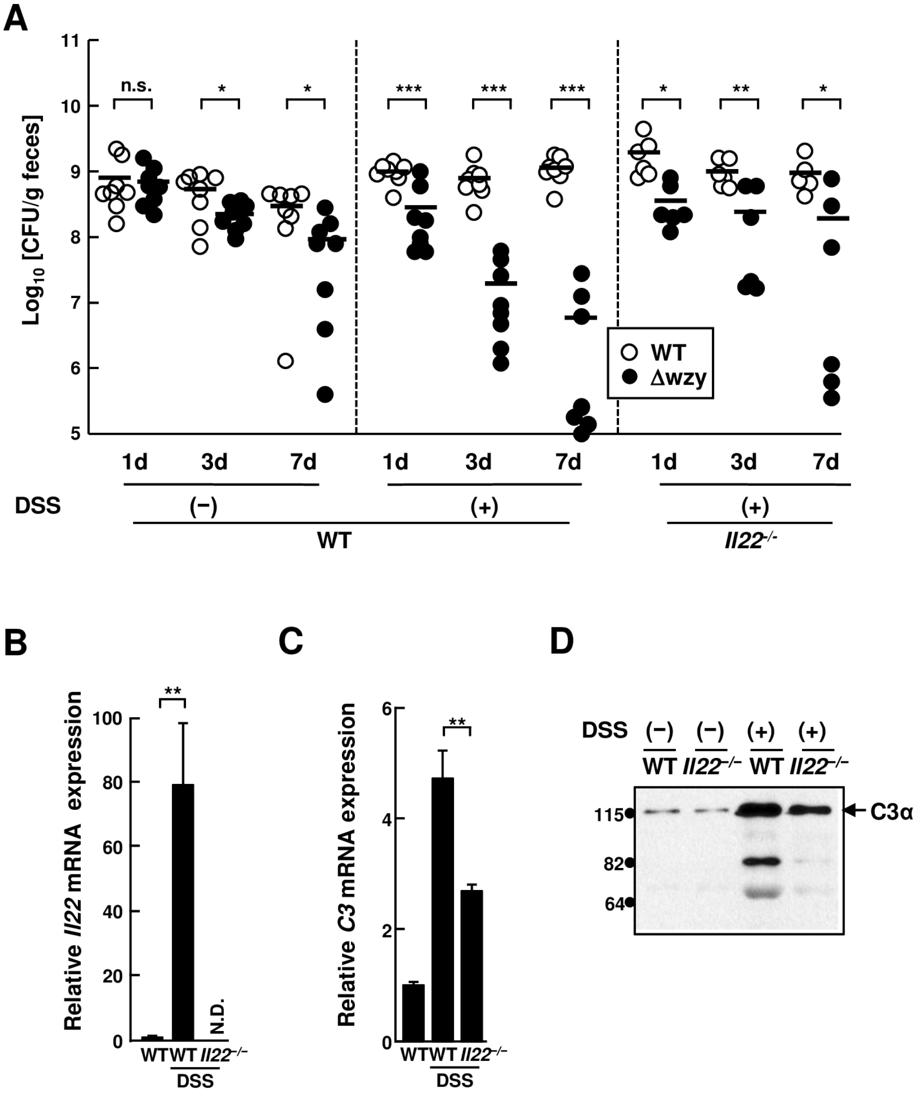

Supplement: S4 Fig — (A) WT and Il22−/−mice were inoculated with a mixture of equal numbers of NI1429Str (WT) and NI1429StrΔwzy::Cm (Δwzy) bacteria after treatment with streptomycin for 1 day, followed by administration of DSS or mock for 7 days and regular water for 1 day. The numbers of WT and the wzy mutant bacteria were determined by plating at the indicated time points. The results from this experiment correspond to those shown in Fig 3E. (B) Relative expression levels of Il22 mRNA in the colon of WT and Il22−/− mice treated with or without DSS. N. D., not detected. (C) mRNA expression levels of C3 in the liver of WT mice, DSS-treated WT mice, and DSS-treated Il22−/− mice. (n = 5–6) (D) C3 levels in serum of WT mice, DSS-treated WT mice, and DSS-treated Il22−/− mice. WT; NI1429Str, Δwzy; NI1429StrΔwzy. Error bars represent SEM. *p < .05, **p < .01. (PDF) [file ppat.1008928.s004.pdf]
