# Supplementary material for: Lipopolysaccharide O structure of adherent and invasive Escherichia coli regulates intestinal inflammation via complement C3
Source: PLoS Pathog. 2020 Oct 7;16(10):e1008928. doi: 10.1371/journal.ppat.1008928 (PMC7571687; doi:10.1371/journal.ppat.1008928)

**S5 Fig. Decreased bacterial loads of the *wzy* AIEC mutant in the mesenteric lymph nodes after DSS treatment**

(A) Individual parameters of histological scores shown in Fig. 4E. (B)The numbers of tested bacteria in mesenteric lymph node (MLN) and liver after 7 days of DSS treatment and 1 day of regular water. Error bars represent SEM. *$p < .05$

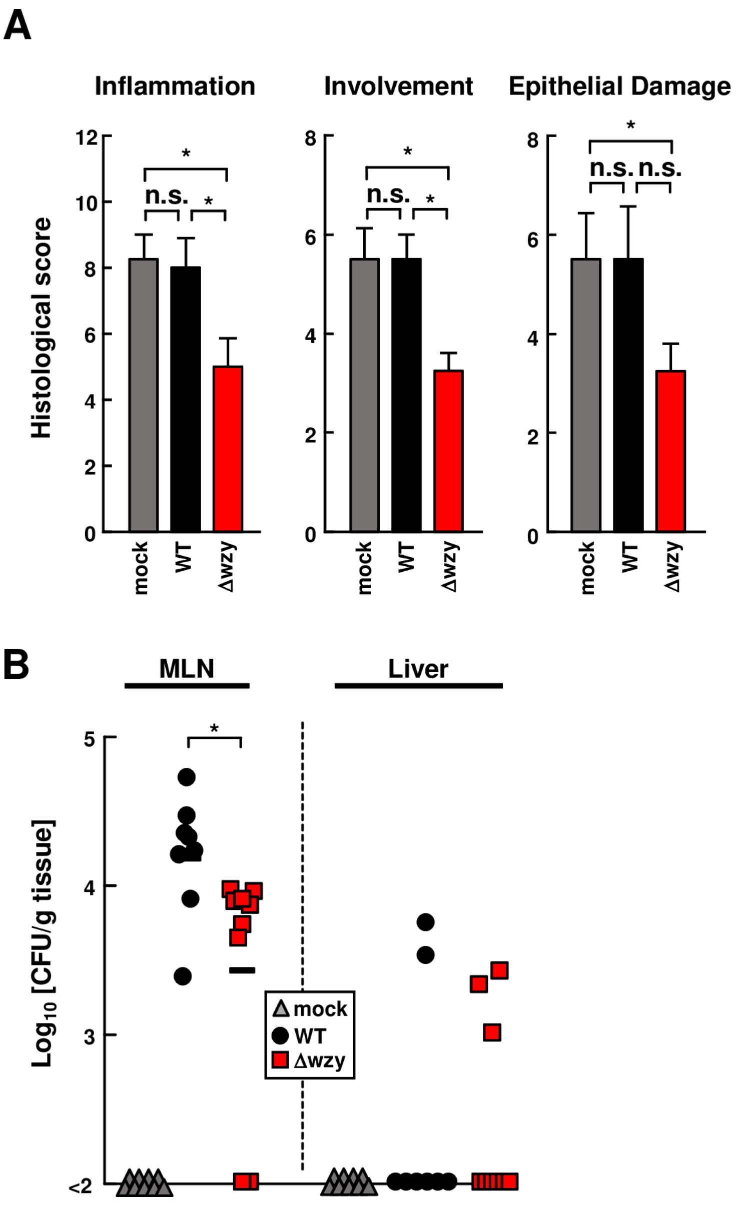

Supplement: S5 Fig — (A) Individual parameters of histological scores shown in Fig 4E. (B)The numbers of tested bacteria in mesenteric lymph node (MLN) and liver after 7 days of DSS treatment and 1 day of regular water. Error bars represent SEM. *p < .05. (PDF) [file ppat.1008928.s005.pdf]
