# Supplementary material for: Lipopolysaccharide O structure of adherent and invasive Escherichia coli regulates intestinal inflammation via complement C3
Source: PLoS Pathog. 2020 Oct 7;16(10):e1008928. doi: 10.1371/journal.ppat.1008928 (PMC7571687; doi:10.1371/journal.ppat.1008928)

S6 Fig. Similar numbers of macrophages, neutrophils and dendritic cells in the intestinal tissue of WT- and Δ*wzy*-colonized mice after 7 days of DSS treatment.

Absolute numbers of macrophages (CD45+ CD11b+ MHC II+ Ly6C-), monocytes (CD45+ CD11b+ LY6C+ MHC II-), DCs (CD45+ CD11c+ MHC II+) and neutrophils (CD45+ CD11b+ Ly6G+) in the colon (n = 4-5)

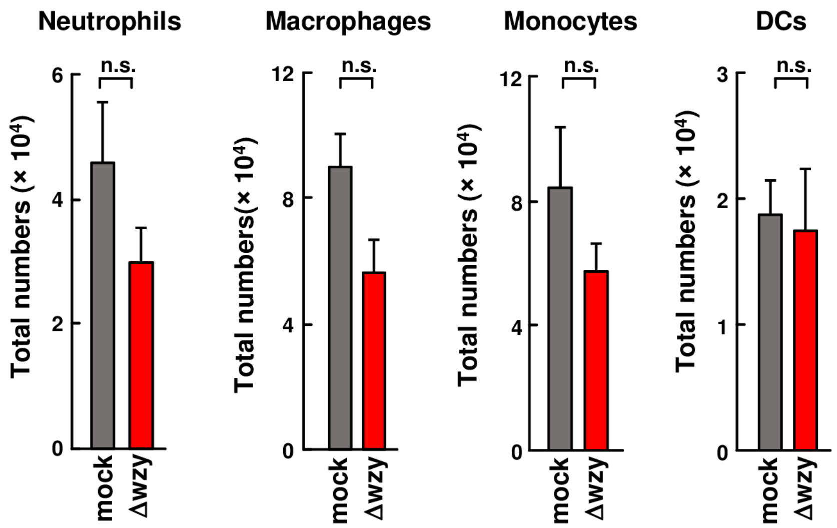

Supplement: S6 Fig — Similar numbers of macrophages, neutrophils and dendritic cells in the intestinal tissue of WT- and Δwzy-colonized mice after 7 days of DSS treatment. Absolute numbers of macrophages (CD45+ CD11b+ MHC II+ Ly6C-), monocytes (CD45+ CD11b+ LY6C+ MHC II-), DCs (CD45+ CD11c+ MHC II+) and neutrophils (CD45+ CD11b+ Ly6G+) in the colon (n = 4–5). (PDF) [file ppat.1008928.s006.pdf]
