# Supplementary material for: Lipopolysaccharide O structure of adherent and invasive Escherichia coli regulates intestinal inflammation via complement C3
Source: PLoS Pathog. 2020 Oct 7;16(10):e1008928. doi: 10.1371/journal.ppat.1008928 (PMC7571687; doi:10.1371/journal.ppat.1008928)

S7 Fig. C3 depletion and engulfment activity after treatment with CVF, and detailed histological scores shown in Fig. 5.

(A) Sera were collected from WT mice treated intraperitoneally with 25 μg/body of cobra venom factor or mock. Serum C3 was determined by immunoblotting. (B) Bone marrow macrophages were incubated with the indicated bacterial strains with or without 5% sera from WT mice treated with 25 μg/body of cobra venom factor or mock. Internalized bacteria were counted after treatment with gentamicin (n =3). (C) Individual parameters of histological scores shown in Fig. 5C. Data represent pooled results from two independent experiments. Error bars represent SEM. ***$p < .001$

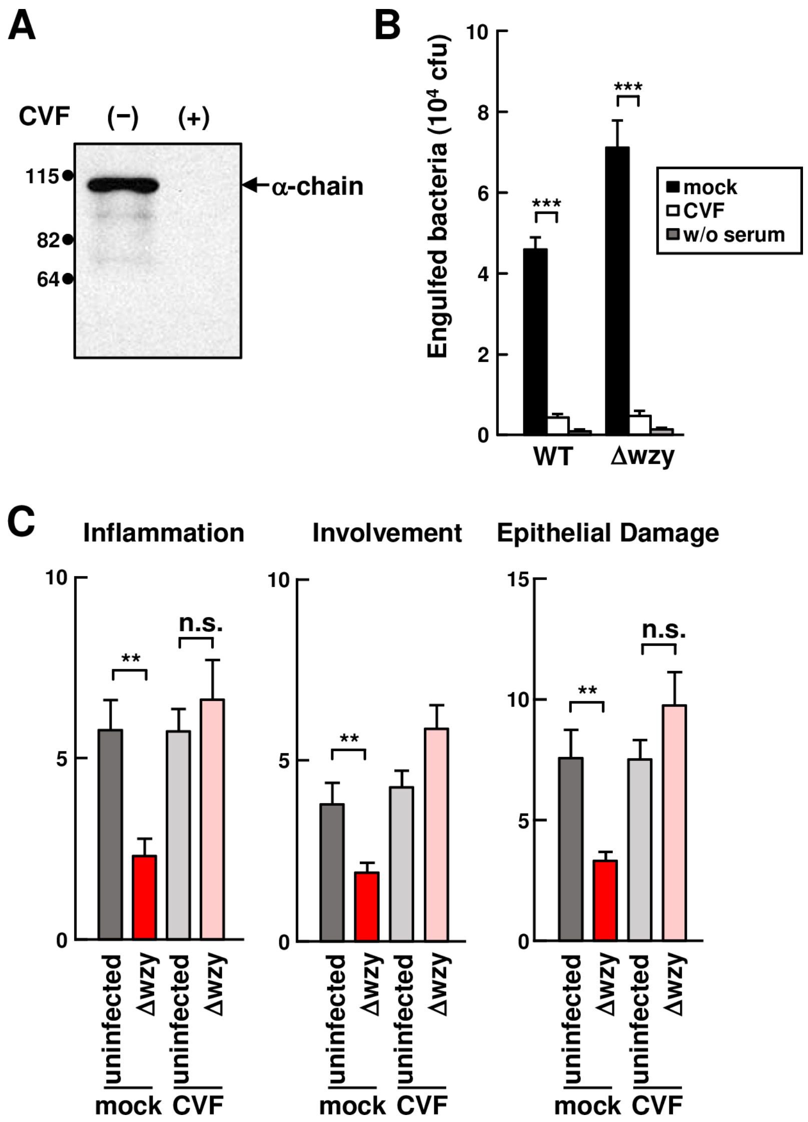

Supplement: S7 Fig — (A) Sera were collected from WT mice treated intraperitoneally with 25 μg/body of cobra venom factor or mock. Serum C3 was determined by immunoblotting. (B) Bone marrow macrophages were incubated with the indicated bacterial strains with or without 5% sera from WT mice treated with 25 μg/body of cobra venom factor or mock. Internalized bacteria were counted after treatment with gentamicin (n = 3). (C) Individual parameters of histological scores shown in Fig 5C. Data represent pooled results from two independent experiments. Error bars represent SEM. ***p < .001. (PDF) [file ppat.1008928.s007.pdf]
