# Supplementary material for: Lipopolysaccharide O structure of adherent and invasive Escherichia coli regulates intestinal inflammation via complement C3
Source: PLoS Pathog. 2020 Oct 7;16(10):e1008928. doi: 10.1371/journal.ppat.1008928 (PMC7571687; doi:10.1371/journal.ppat.1008928)
Supplement: S2 Table — (DOCX) [file ppat.1008928.s009.docx]

**S2 Table, Gene orthlogue groups found in E. coli strains.**

**To download S2 Table,**

**Please access**

[**http://www-personal.umich.edu/~ino/S2 Table.zip**](http://www-personal.umich.edu/~ino/S2%20Table.zip)

**(zipped file 19,175,590 bytes; original size 86,071,518 bytes)**
