# Supplementary material for: Lipopolysaccharide O structure of adherent and invasive Escherichia coli regulates intestinal inflammation via complement C3
Source: PLoS Pathog. 2020 Oct 7;16(10):e1008928. doi: 10.1371/journal.ppat.1008928 (PMC7571687; doi:10.1371/journal.ppat.1008928)
Supplement: S3 Table — (DOCX) [file ppat.1008928.s010.docx]

**S3 Table. *E. coli* strains and plasmids used in this study**

Strains and plasmids Description Source and reference

***E. coli***

K-12 substr. MG1655 Non-pathogenic commensal. Complement-sensitive control ATCC

NI1076 Mouse ExPEC strain isolated from liver of *Il22^−/−^* mice infected with [1]

*Clostridium difficile*.

LF82 AIEC reference strain [2]

NI1396 isolate from feces of *Il22^−/−^* mice 7 days after DSS-treatment This study

NI1413 isolate from feces of *Il22^−/−^* mice 7 days after DSS-treatment This study

NI1423 isolate from feces of *Il22^−/−^* mice 7 days after DSS-treatment This study

NI1522 isolate from feces of *Il22^−/−^* mice 7 days after DSS-treatment This study

NI1429 isolate from feces of *Il22^−/−^* mice 7 days after DSS-treatment This study

NI1429Str Str^r^ strain of NI1429 This study

NI1429StrΔ*wzy*::Cm NI1429str with *wzy* gene replaced with CAT cassette This study

NI1429StrΔ*wzy* NI1429str with *wzy* gene deleted This study

NI1429StrΔwzy[pGEM-T-w*zy*] complemented strain of NI1429str Δ*wzy* This study

NI1429strΔ*fimH*::Cm NI1429str with *fimH* gene replaced with CAT This study

NI1429strΔ*fimH* NI1429str with *fimH* gene deleted This study

NI1429Str[pNI1429Amp] NI1429str carrying pNI1429Amp This study

NI1429Str[pNI1429AmpΔcol] NI1429str carrying pNI1429AmpΔcol This study

NI1159 isolate from feces of *C. difficile*-infected *Il22*^-/-^ mice [1]

NI1163 isolate from feces of *C. difficile*-infected *Il22*^-/-^ mice [1]

NI1165 isolate from feces of *C. difficile*-infected *Il22*^-/-^ mice [1]

NI491 mouse commensal strain [1]

NI491Str Str^r^ strain of NI491 [1]

**Plasmids**

pKD3 Plasmid with FRT-flanked chloramphenicol-resistant gene [3]

pKD46 Plasmid expressing λRed recombinase system [3]

pCP20 Plasmid expressing FLP recombinase [3]

pGEM-T-*wzy* pGEM-T carrying a 10.7 kb fragment of *wzy* gene of NI1429 This study

pNI1429 Endogenous plasmid in NI1429 This study

pNI1429Amp Plasmid carrying ampicillin-resistant gene This study

pNI1429AmpΔcol Colicin Y deletion mutant of the plasmid pNI1429 This study

References:

1. Hasegawa M, Yada S, Liu MZ, Kamada N, Munoz-Planillo R, Do N, et al. Interleukin-22 regulates the complement system to promote resistance against pathobionts after pathogen-induced intestinal damage. Immunity. 2014;41(4):620-32. doi: 10.1016/j.immuni.2014.09.010. PubMed PMID: 25367575; PubMed Central PMCID: PMCPMC4220303.

2. Darfeuille-Michaud A, Boudeau J, Bulois P, Neut C, Glasser AL, Barnich N, et al. High prevalence of adherent-invasive Escherichia coli associated with ileal mucosa in Crohn's disease. Gastroenterology. 2004;127(2):412-21. PubMed PMID: 15300573.

3. Datsenko KA, Wanner BL. One-step inactivation of chromosomal genes in Escherichia coli K-12 using PCR products. Proc Natl Acad Sci U S A. 2000;97(12):6640-5. doi: 10.1073/pnas.120163297. PubMed PMID: 10829079; PubMed Central PMCID: PMCPMC18686.
