# Supplementary material for: Lipopolysaccharide O structure of adherent and invasive Escherichia coli regulates intestinal inflammation via complement C3
Source: PLoS Pathog. 2020 Oct 7;16(10):e1008928. doi: 10.1371/journal.ppat.1008928 (PMC7571687; doi:10.1371/journal.ppat.1008928)
Supplement: S4 Table — (DOCX) [file ppat.1008928.s011.docx]

**S4 Table. PCR primers used in this study**

Primer Purpose Sequence

1429WzyD5 Mutagenesis AAAAAAATGCTTATGGTCAGTACTTAATCAAATTGACACAGGGATAATAAgtgtaggctg

gagctgcttc

1429WzyD3 Mutagenesis TTCTGAGGTAGATCTCCGTTATAGACACTCATAATAACAGCTATCTTGTCcatatgaatat

cctcctta

D-Eco1429-FimH Fw Mutagenesis ACAGCTGAACCCGAAGAGATGATTGTAATGAAACGAGTTATTACCCTGTTTGCTgtaggc

tggagctgcttcg

D-Eco1429-FimH Rv Mutagenesis CAGCATTAGCAATGTCCTGTGATTTCTTTATTGATAAACAAAAGTCACGCCAATcatatga

atatcctccttag

1429WzyC5 Genotyping AATTGAAGAGCGTCAGGGGC

1429WzyC-WT3 Genotyping GTTGCCAGTGCTTGCCTTAC

CmCassetteC3 Genotyping GGCAATGAAAGACGGTGAGC

FimH common 5 Genotyping CATTCAGGCAGTGATTAGCATC

FimH WT 3 Genotyping GATAACACGCCGCCATAAGC

FimH-Cm mutant 3 Genotyping GGCGTGTTACGGTGAAAACC

WzyCompS Complementation TGATATCAGGGATAATAAATGGAATCTCTA

WzyCompA Complementation GTCGACTTATACCTCGGTATCTAAATAAATG

Amp5Bam Plasmid construction GGGGATCCGGAGATCTCCCGATCCGTC

Amp3XhoI Plasmid construction CCGGGAATTCTAGAAGATCCTTTGATCTTTTCT

Y5Bam Plasmid genotyping AAGGATCCTGGTGTTGGTCACCTCTGGA

Y3Xba Plasmid genotyping AACCCGGGTCTAGATTCGCTGTGTCCACATC

ColY5 ColY genotyping GCTTCTGTCAATAAACTGATGGCTAATC

ColY3 ColY genotyping AAACTTATTCCCCATATCCTCTGCATCA

UniF340 qPCR ACTCCTACGGGAGGCAGCAGT

UniR514 qPCR ATTACCGCGGCTGCTGGC

Eco1457F qPCR CATTGACGTTACCCGCAGAAGAAGC

Eco1652R qPCR CTCTACGAGACTCAAGCTTGC

mGapdhS qPCR CCCTTAAGAGGGATGCTGCC

mGapdhA qPCR TACGGCCAAATCCGTTCACA

mIL-22S qPCR GCTCAGCTCCTGTCACATCA

mIL-22A qPCR AGCTTCTTCTCGCTCAGACG

mC3S qPCR GGGCTGTTAAATGGTTGATTCTG

mC3A qPCR GATGAGGACGAAGGCTGTG
